# Identifying the demographic pathways linking environmental covariates to population dynamics in an avian migrant

**DOI:** 10.1002/eap.70166

**Published:** 2026-01-05

**Authors:** Ellen C. Martin, Thomas V. Riecke, Pierre‐Alain Ravussin, Daniel Arrigo, Michael Schaub

**Affiliations:** ^1^ Swiss Ornithological Institute Sempach Switzerland; ^2^ University of Montana Missoula Montana USA; ^3^ Rue du Theu 12 Baulmes Switzerland; ^4^ Hofmattenstrasse 12 Nidau Switzerland

**Keywords:** avian ecology, breeding ground environment, demography, European pied flycatcher, *Ficedula hypoleuca*, integrated population model

## Abstract

Understanding and predicting the effects of climate change on populations requires linking the environmental conditions to demographic rates and the demographic rates to population‐level consequences, but often this complete demographic pathway is not studied. Integrated population models (IPMs) incorporate demographic data into a single analytical framework, allowing for the inclusion of environmental covariates to test hypotheses considering how the environment influences demographic rates, and consequently, to which demographic rates population growth rate is most sensitive. In birds, there is strong evidence that environmental conditions impact population growth, and that long‐distance migrant avian species with short phenological windows are at greatest risk of population decline due to changing environmental conditions. We built a Bayesian IPM with over 40 years of mark‐recapture, fecundity, and nest box occupancy data and incorporated environmental covariates hypothesized to be driving the observed changes in two populations of a fast‐lived long‐distance migrant, the European pied flycatcher. Using variance decomposition methods, we identified the demographic pathways through which environmental covariates were acting. While several environmental covariates impacted fecundity and survival, only precipitation acted via apparent juvenile and adult survival contributed significantly to variation in population growth rate. Increased precipitation during the nest initiation, incubation, and hatchling stages had negative carry‐over effects on juvenile survival during the post‐fledging and overwintering period, and increased precipitation negatively impacted adult apparent survival, likely due to the increased energetic demands of caring for eggs and hatchlings in challenging conditions and reduced availability of aerial prey. We show that linking environmental covariates to demographic rates does not sufficiently explain or predict population‐level consequences, and that decomposing variation along the complete demographic pathway is a necessary step to appropriately identify how covariates influence population dynamics.

## INTRODUCTION

Accurately estimating demographic rates and population abundance is a fundamental goal of population ecology and necessary for sound wildlife conservation and management in the face of climate change (Williams et al., 2002). Tracking these rates and population sizes through time can allow for the identification of drivers of changes in demographic rates and is useful when determining specific population processes limiting population growth (Turchin, 2013). Studies on population dynamics across taxa reveal strong links between environmental covariates, specifically temperature and precipitation, and demographic rates such as survival, fecundity, and recruitment (Parmesan & Yohe, 2003; Pearce‐Higgins et al., 2015). In birds, environmental covariates have been shown to independently and synergistically influence survival (Newton, 2007) and fecundity (Halupka et al., 2023). For example, rainfall can negatively impact juvenile survival in passerines (Grüebler & Naef‐Daenzer, 2010), and there is evidence, again in passerines, showing lower fecundity and recruitment after rainfall events coupled with low temperatures (Siikamäki, 1996; Veistola et al., 1997). The influence of temporal variation in environmental covariates on demographic rates such as survival and fecundity is expected to change in complex ways due to climate change (IPCC, 2023).

The influence of environmental conditions can scale up to population growth when its effects on demographic rates align with those to which population growth is most sensitive (Jenouvrier et al., 2010; Knape et al., 2023). Historically, demographic rates have been analyzed in isolation from one another, limiting our ability to identify how environmental effects work through multiple demographic pathways. Population dynamics are the culmination of multiple demographic processes, and the same environmental condition may simultaneously influence several demographic rates. Investigating these demographic rates in isolation poses challenges for comparisons across demographic rates and disentangling the relative contributions of each to population change (Koons et al., 2016; Schaub & Abadi, 2011). Importantly, not all demographic rates contribute equally to population growth. Some rates may be labile, or highly variable in response to environmental conditions, but have low sensitivity, meaning that changes in these rates have limited influence on population growth, whereas rates that most strongly influence population growth tend to vary little over time, a pattern consistent with demographic buffering that reduces vulnerability to environmental fluctuations (Hilde et al., 2020; Koons et al., 2009). Understanding how variation in environmental conditions scale up through multiple demographic pathways to influence population growth rates is essential for predicting species responses to climate change, and to guide conservation strategies.

Identifying these demographic pathways is particularly important for species of conservation concern, where understanding the mechanisms of population change is critical for targeting conservation efforts. Long‐distance migrant avian species with narrow breeding windows are thought to be at greatest risk of population declines caused by changing environmental conditions, as their annual cycles limit opportunities to recover from poor conditions and carry‐over effects, in which conditions experienced during one part of the annual cycle influence demographic rates in subsequent seasons (Both et al., 2006). Carry‐over effects are well documented in migratory birds and can amplify environmental impacts (Both et al., 2017). In such systems, scaling environmental variation to the population level requires integrating demographic and environmental data across breeding, migration, and wintering periods.

IPMs allow for the inclusion of environmental covariates to test hypotheses related to their relative influence on different demographic rates (Schaub & Kéry, 2022). Using an IPM allows us to quantify how temporal variation in each demographic rate contributes to population dynamics and how much the variation of an environmental covariate has contributed (Knape et al., 2023; Koons et al., 2016, 2017). Using a variance decomposition analysis like that developed by Knape et al. (2023), we can infer spatial and temporal patterns that drive population dynamics. For example, we can identify the time and space in the annual cycle of a species that are most responsible for driving population dynamics. This allows us to understand population trends through time, predict change into the future under changing environmental conditions, and appropriately allocate conservation resources.

The European pied flycatcher (hereafter “pied flycatcher”; *Ficedula hypoleuca*) is one of Europe's most extensively studied passerines (Lundberg & Alatalo, 2010; Sanz, 2001), and is a model candidate through which to examine the impacts of environmental changes on population dynamics (Both & Visser, 2001). The pied flycatcher has experienced regional population declines across parts of its range, likely driven by shifts in environmental conditions across its annual cycle (Both et al., 2006). In fast‐lived passerines like pied flycatchers, population growth is typically most sensitive to variation in fecundity and first‐year survival (Sæther & Bakke, 2000) but linking environmental conditions to these pan‐European declines via demographic rates is rare. Despite extensive pan‐European mark‐recapture studies and research on fecundity components, estimates of apparent survival (defined as the product of the probabilities of true survival and of site fidelity) are still infrequent (Ravussin et al., 2007; Sanz, 2001), and only one study has used an IPM framework to examine ecological drivers of pied flycatcher population dynamics (Nater et al., 2023). There remain substantial gaps in our understanding of how environmental conditions influence population dynamics across multiple demographic rates and life stages for the pied flycatcher.

Here, we identify temporal trends in the demographic rates and population abundances of two pied flycatcher populations breeding in Switzerland over 40 years. We built a Bayesian IPM and integrated mark‐recapture, fecundity, and nest box count data with environmental (temperature, precipitation, Normalized Difference Vegetation Index [NDVI], tree mast years) and phenological (nest initiation date) covariates hypothesized to be impacting demographic rates to understand population‐level responses to changing ecological conditions and uncover drivers of changes in population growth rates. These covariates encompass both direct and indirect pathways through which climate change may affect vital rates by reflecting environmental conditions, prey dynamics, food availability, or phenological mismatches on breeding and nonbreeding grounds. Following methods developed by Knape et al. (2023), we decomposed the temporal variation of population growth into contributions from each demographic rate, whose temporal variation may partially be due to variation in the considered covariates. This allowed for the estimation of the relative contributions of each environmental or phenological covariate to changes in the population growth rates and the identification of the underlying demographic pathway. Knape et al. (2023) demonstrated that even with detailed demographic and environmental data, roughly half the variation in population growth remained unexplained, highlighting the challenges of linking environmental variability to population‐level outcomes. Here, we apply a variance decomposition approach, inspired by their work, to a different species and ecological context, with the goal of evaluating how key environmental covariates contribute to variation in demographic rates and ultimately to population growth rates. We hypothesized that local environmental and phenological variables would influence population growth rates in this fast‐lived passerine by acting on female juvenile survival and fecundity, which population growth rate tends to be sensitive to in fast‐lived species. By applying an IPM framework to two Swiss populations of pied flycatchers, we demonstrate how a migratory bird responds demographically to changing ecological conditions, and the concomitant impact these changing demographic rates have on population dynamics. This allowed us to determine which covariates and time periods had the greatest influence on population dynamics and offers a framework for disentangling complex demographic responses to environmental change in other migratory species.

## MATERIALS AND METHODS

### Study species

Pied flycatchers are small (~13 g), short‐lived (average lifespan of 6 years; Herényi et al., 2012), cavity‐nesting passerines that inhabit temperate and boreal forests across Europe during the breeding season. They breed primarily in oak‐dominated (*Quercus* spp.) forest at mid‐elevation (~500 m asl), and readily use nest boxes (Lundberg & Alatalo, 2010; Slagsvold, 1987). Reproduction typically begins at one year of age, and they exhibit biparental care of their single brood containing between five to seven eggs (Lundberg & Alatalo, 2010). Adults exhibit a high degree of philopatry, with occasional short‐distance (i.e., <1 km) breeding dispersal movements (Chernetsov et al., 2006).

**FIGURE 1:**
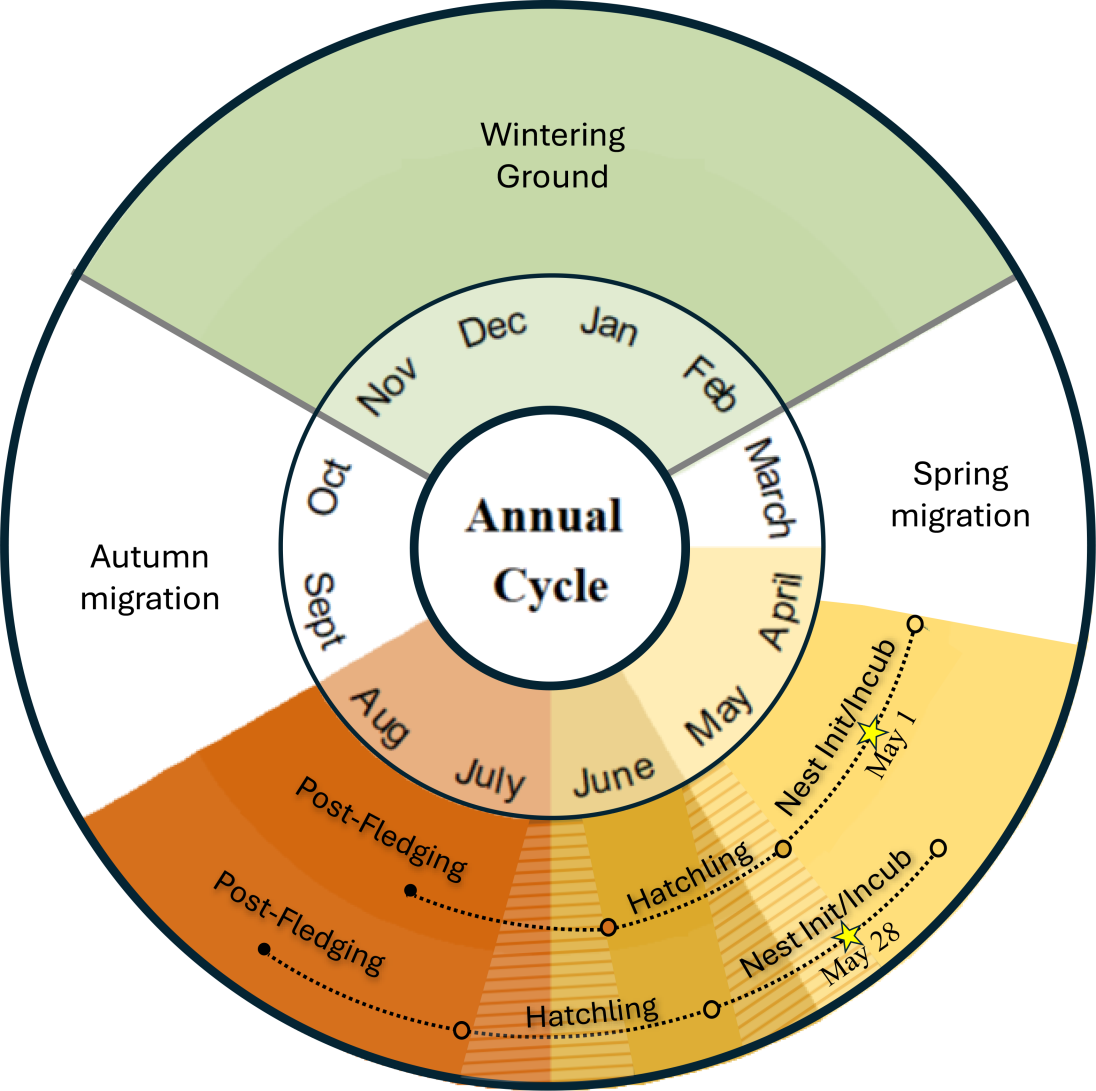
Major life cycle periods in the annual cycle of the migratory pied flycatcher. It begins in January on the wintering grounds from which birds depart at the end of February. They migrate during March and begin arriving at breeding grounds in April. Nest initiation/incubation (“Nest Init/Incub”) starts in April and continues through mid‐to‐late May. Birds are in the “Hatchling” period for 16 days, afterwards in the post‐fledging period (“Post‐Fledging”) until the end of August. They migrate in early September and arrive on their wintering grounds at the beginning of November (“Wintering Ground”). Solid gray lines between periods indicate that the dates did not vary annually and were fixed based on a literature review. Nest initiation/incubation, hatching, and post‐fledging periods were calculated in relation to the nest initiation date available for each population and year. The two example dates (designated with stars) show the two extremes: May 1st was the earliest, while May 28th was the latest average mean nest initiation date. Colors correspond to periods, and circles designate the start date of each period. The color‐banded pattern over two colors indicates that the dates could belong to either of the periods on either side of the color bands, depending on the nest initiation date.

Pied flycatchers are long‐distance migrants that spend approximately 4 months on their breeding grounds in central Europe, May through early/mid‐August, before migrating to western sub‐Saharan African countries for the non‐breeding season (Figure 1). The exact locations to which pied flycatchers that breed in Switzerland migrate remain unknown (Adamík et al., 2023).

### Study areas

Population‐ and individual‐level data were collected from two nest box breeding populations in the Swiss municipalities of Baulmes (46°47′ N, 6°32′ E) and Corcelles‐près‐Concise (hereafter “Corcelles”; 46°50′ N, 6°42′ E) in the canton of Vaud (Appendix S1). These populations were located ~15 km apart and positioned on the southwestern edge of pied flycatchers' breeding range (Keller et al., 2020). Annual systematic monitoring began in Baulmes and Corcelles in 1980 and 1989 respectively and, while the study is still ongoing, ringing ended in 2020. The methods of this field study have been reviewed by an advisory committee of the Swiss Ringing Centre, and licenses to mark birds have been issued by the Swiss Ringing Centre. Approval has been granted in accordance with the applicable legal requirements.

Baulmes and Corcelles had on average 110 (range: 20–162) and 186 (range: 138–247) nest boxes available annually, with an average annual occupancy of 0.15 (range: 0.07–0.50) and 0.15 (range: 0.06–0.23), respectively (Appendix S2). Given pied flycatchers' known preference for artificial nest boxes compared to natural cavities (Slagsvold, 1987) and the low occupancy rates at each site, we assumed that nest boxes were provided in excess and that we detected a reliable index of breeding pied flycatchers during nest monitoring. Over the course of the study, 641 nests in Baulmes (annual mean = 15.63, SD = 5.38) and 718 nests in Corcelles (annual mean = 25.09, SD = 7.14) were monitored.

### Data collection

Annual nest box monitoring in Baulmes and Corcelles resulted in three primary data types used in our IPM: (1) mark‐recapture data from individual ringing and subsequent recapture to inform apparent survival estimates (see *Mark‐recapture data*), (2) nest success parameters to inform fecundity estimates (see *Reproductive data*), and (3) annual counts of the occupied number of nest boxes per population to inform abundance estimates (see *Abundance data*). See Ravussin et al. (2007) for further details.

#### Mark‐recapture data

During nest box monitoring, we captured and marked juvenile and breeding adult male and female pied flycatchers with unique rings. We primarily captured females that were incubating eggs, whereas, we primarily caught males that were feeding nestlings (Ravussin et al., 2007). Over the 40‐year span of the study, we ringed 3378 individuals at Baulmes and 2976 individuals at Corcelles. Ages were known for individuals that were ringed during their year of birth and subsequently returned to the populations the following year as recruits (*n* = 582). Birds captured for the first time as adults were assumed to be 1‐year‐old immigrant natal dispersers from other populations (*n* = 371). There were a high number of associated breeding recaptures of previously marked individuals (individuals recaptured at least once = 1755).

#### Reproductive data

We checked nests in nest boxes at minimum once a week during the breeding season and recorded data on nest initiation date, clutch size, number of hatchlings, number of fledglings, and female parent ring identification. We excluded nests where females could not be recaptured (*n* = 173 [Baulmes], and *n* = 178 [Corcelles]) from the analysis of fecundity because fecundity parameters were modeled as a function of female stage (i.e., age class). Flycatchers can be polygynous (Ravussin et al., 2024), but we did not account for this in our female‐based population model.

#### Abundance data

We assumed the count of annually occupied nest boxes was a reliable index of the total population size of each sex and therefore used the number of occupied nest boxes at each population as the abundance of females and males.

#### Environmental covariates

We downloaded data on daily temperature means, minimums, maximums, and cumulative precipitation from the closest daily measurement station to the populations (47°0′16, 6°57′21; SwissMetNet, 2024). We summarized daily data into average values for biologically relevant time periods corresponding to pied flycatcher life cycle events on the breeding grounds (Figure 1). These periods varied annually with the mean nest initiation date at each site (Appendix S3).

The nest initiation/incubation period was defined as the time period that began 14 days before the annual mean nest initiation date and ended after the mean number of egg‐laying days (six), plus a 14‐day incubation period, totaling 34 days (Nater et al., 2023). Hatchling period was the 16‐day window that encompassed the average number of pre‐fledging days chicks spent in the nest and began immediately following the nest initiation/incubation period. Finally, the post‐fledging period was the time period from the end of the hatchling period plus 10 days, which was an estimate for departure date for wintering grounds (Lundberg & Alatalo, 2010; Figure 1). We *z*‐standardized all environmental data across years before analysis (Appendix S4: Figure S1, panels A–F).

Nest depredation and nest box occupancy by European edible dormice (*Glis glis*) occurred at the two study sites (e.g., 14 documented cases of known nest predation by dormice during the study period). Dormice are common arboreal small rodents that breed in tree cavities and nest boxes and reproduce in years with beech (*Fagus* spp.) or oak (*Quercus* spp.) seeding (Pilastro et al., 2003). They are detrimental to cavity‐nesting birds such as pied flycatchers because they depredate eggs and hatchlings during the late summer as well as occupy nest boxes (Adamík & Král, 2008). Dormouse nest depredation of avian cavity‐nesting birds is a known and well‐documented phenomenon across species, with higher‐than‐average rates of depredation in migratory flycatcher species (Adamík & Král, 2008). Identity of flycatcher nest predators is difficult to assign (Lima, 2002), making dormouse predation difficult to attribute as the cause of nest failure or juvenile death without dedicated camera traps or video surveillance (e.g., Adamík & Weidinger, 2024). Given this difficulty, we expect that nest depredation in our system happened more frequently than the documented 14 cases and could have played an important role in driving survival dynamics. We used mast years of oak trees as a proxy for dormouse abundance which were available for the years 1988 until 2020 from the Swiss Federal Research Institute MastWeb application (Wald, Schnee, und Landschaft (WSL) MastWeb Application, n.d.) based on masting activity of the closest oak tree to the study site each year to determine if there were negative effects of dormice on reproductive parameters (Appendix S4: Figure S1, panel G). Oak trees produce a mast crop in Switzerland in September or October, and there is evidence that dormice anticipate these events and increase reproductive rates in the spring of years that will have mast fruiting trees (Lebl et al., 2011). This would result in both a high abundance of juvenile dormice in the year of the mast event (the anticipatory increase in reproduction), and in the following year as a high abundance of dormice given that overwinter survival is extremely high (Pilastro et al., 2003).

To explore the effects of wintering ground environmental conditions as carry‐over effects on parameters estimated on the breeding grounds the following year, we represented the environmental conditions pied flycatchers experienced on their overwintering grounds using the NDVI data (PKU GIMMS NDVI, version 1.2) collected at half‐month intervals and 1/12° resolution (Li et al., 2023) for sub‐Saharan Western African countries (i.e., Benin, Burkina Faso, Cape Verde, Côte d'Ivoire, The Gambia, Ghana, Guinea, Guinea‐Bissau, Liberia, Nigeria, Senegal, Sierra Leone, Togo, and Cameroon). Due to the broad spatial and temporal scale of the non‐breeding season and the absence of precise location data, NDVI used in this analysis should be interpreted as a general proxy for large‐scale environmental productivity, rather than as an exact measure of local conditions experienced by individuals. We calculated the mean value and *z*‐standardized all measurements taken between November 1 and February 29 in each non‐breeding season (Appendix S4: Figure S1, panel H). We checked correlations between all environmental covariates (Appendix S5) and used a correlation threshold of >|0.50| to exclude correlated covariates from the same model.

### Data analysis

#### The integrated population model

We constructed a stochastic stage‐structured IPM to study the population dynamics of pied flycatchers (Appendix S6). Model parameters varied through time and across the two sites and were defined by stage (ad: adults, juv: juveniles) and sex classes (f: females, m: males) for apparent survival, and stage (ad: adults, im: immigrants, rec: recruits) classes for fecundity parameters. Additional details on parameters and indexing are available in Appendix S7.

The total population $\left(N_{s,t}\right)$ at site *s* and year *t* was composed of local recruits (1 year old and locally born; $N_{j,\mathrm{rec},s,t}$), adults (2 years old or older; $N_{j,\mathrm{ad},s,t}$) and immigrants (assumed 1 year old and not locally born; $N_{j,\mathrm{im},s,t}$) of each sex *j*.

Using a pre‐breeding census, the population dynamics of females at each site *s* from year *t* to *t* + 1 were described with the following stage‐specific equations:

(1) $$N_{f,s,t+1}=N_{f,\mathrm{rec},s,t+1}+N_{f,\mathrm{ad},s,t+1}+N_{f,\mathrm{im},s,t+1}$$

(2) $$\begin{array}{l} N_{f,\mathrm{rec},s,t+1}˜\text{Poisson}\left(0.5\phi_{f,\mathrm{juv},s,t}\left(N_{f,\mathrm{rec},s,t}\kappa_{\mathrm{rec},s,t}\zeta_{\mathrm{rec},s,t}\right.\right.\\ \;\left.\left.+N_{f,\mathrm{ad},s,t}\kappa_{\mathrm{ad},s,t}\zeta_{\mathrm{ad},s,t}+N_{f,\mathrm{im},s,t}\kappa_{\mathrm{im},s,t}\zeta_{\mathrm{im},s,t}\right)\right) \end{array}$$

(3) $$N_{f,\mathrm{ad},s,t+1}˜\text{binomial}\left(N_{f,\mathrm{rec},s,t}+N_{f,\mathrm{ad},s,t}+N_{f,\mathrm{im},s,t},\phi_{f,\mathrm{ad},s,t}\right)$$

(4) $$N_{f,\mathrm{im},s,t+1}˜\text{Poisson}\left(\omega_{f,s}\right).$$

The number of local recruits was a function of the number of female recruits, female adults, and female immigrants that reproduced during year *t* with stage‐varying clutch sizes ($\kappa_{\mathrm{rec},s,t},\kappa_{\mathrm{ad},s,t},\kappa_{\mathrm{im},s,t}$), stage‐varying probabilities of fledging ($\zeta_{\mathrm{rec},s,t},\zeta_{\mathrm{ad},s,t},\zeta_{\mathrm{im},s,t}$), and the probability of juvenile apparent survival from year *t* to *t* + 1 ($\phi_{f,\mathrm{juv},s,t}$). The number of adults in year *t* + 1 was composed of the number of recruits, adults, and immigrants from year *t* that survived to *t* + 1 with adult apparent survival probability $\left(\phi_{f,\mathrm{ad},s,t}\right)$. Finally, the expected number of immigrants in year *t* + 1 was $\omega_{f,s}$ (see *Estimation of immigrants*). To account for demographic stochasticity, we used Poisson and Binomial distributions.

For males, population dynamics at each site *s* from year *t* to *t* + 1 were described with similar stage‐specific equations:

(5) $$N_{m,s,t+1}=N_{m,\mathrm{rec},s,t+1}+N_{m,\mathrm{ad},s,t+1}+N_{m,\mathrm{im},s,t+1}$$

(6) $$\begin{array}{l} N_{m,\mathrm{rec},s,t+1}˜\text{Poisson}\left(0.5\phi_{m,\mathrm{juv},s,t}\;\left(N_{f,\mathrm{rec},s,t}\kappa_{\mathrm{rec},s,t}\zeta_{\mathrm{rec},s,t}\right.\right.\\ \;\left.\left.+N_{f,\mathrm{ad},s,t}\kappa_{\mathrm{ad},s,t}\zeta_{\mathrm{ad},s,t}+N_{f,\mathrm{im},s,t}\kappa_{\mathrm{im},s,t}\zeta_{\mathrm{im},s,t}\right)\right) \end{array}$$

(7) $$N_{m,\mathrm{ad},s,t+1}˜\text{binomial}\left(N_{m,\mathrm{rec},s,t}+N_{m,\mathrm{ad},s,t}+N_{m,\mathrm{im},s,t},\phi_{m,\mathrm{ad},s,t}\right)$$

(8) $$N_{m,\mathrm{im},s,t+1}˜\text{Poisson}\left(\omega_{m,s}\right).$$

The demographic parameters were the same as those used for the females with the exceptions that apparent survival ($\phi_{m,\mathrm{juv},s,t}$, $\phi_{m,\mathrm{ad},s,t}$) and number of immigrants ($\omega_{m,s}$) for males were used. We assumed that the sex ratio of fledglings was even. In the following text, we describe the likelihoods for each dataset integrated into the population model.

#### Estimation of apparent survival probabilities

To estimate probabilities of apparent survival and of recapture, we specified a stage‐structured Cormack–Jolly–Seber model with multinomial likelihood using the mark‐recapture data (Cormack, 1964; Jolly, 1965; Lebreton et al., 1992; Seber, 1965). The apparent survival parameter ($\phi_{j,a,s,t}$) described the probability that an individual of sex *j* and stage class *a* survived from one breeding season to the next and returned to site *s*. We specified sex, stage, site, and year‐dependent recapture probabilities for juveniles and adults ($p_{j,a,s,t}$), which were composed of a sex‐, stage‐ and site‐specific mean ($\mu_{p,j,a,s}$) with variation around the mean ($\varepsilon_{p,j,a,s,t}$),

(9) $$\text{logit}\left(p_{j,a,s,t}\right)=\mu_{p,j,a,s}+\varepsilon_{p,j,a,s,t},\;\text{where}$$

(10) $$\varepsilon_{p,j,a,s,t}˜\text{normal}\left(0,\;\sigma_{p,j,a,s}^{2}\right).$$

Apparent survival was a function of a sex‐, stage‐, and site‐specific mean ($\mu_{\phi ,j,a,s}$), with variation around the mean ($\varepsilon_{\phi ,j,a,s,t}$). The dynamics of apparent survival were described as,

(11) $$\text{logit}\left(\phi_{j,a,s,t}\right)=\mu_{\phi ,j,a,s}+\varepsilon_{\phi ,j,a,s,t},\text{where}$$

(12) $$\varepsilon_{\phi ,j,a,s,t}˜\text{normal}\left(0,\;\sigma_{\phi ,j,a,s}^{2}\right).$$

We accounted for among‐year variation in all apparent survival parameters using environmental covariates that were included as effects using a generalized linear model, allowing for a specific slope parameter for each covariate:

(13) $$\text{logit}\left(\phi_{j,a,s,t}\right)=\mu_{\phi ,j,a,s}+\beta_{\phi ,\mathrm{cov},a}{\text{Covariate}}_{t}+\varepsilon_{\phi ,j,a,s,t},$$

where $\beta_{\phi ,\mathrm{cov},a}$ was an estimated parameter for the slope of the effect of the covariate cov for stage class *a*, and ${\text{Covariate}}_{t}$ was the observed covariate value in year *t*. The covariates we were interested in testing had varying degrees of correlation, with some highly correlated (Appendix S5). We, therefore, ran separate IPMs for each covariate of interest to determine which covariate had a significant influence on survival. As covariates we used the effect of mean, minimum, and maximum daily temperature averages and cumulative precipitation for the time periods corresponding to nest initiation/incubation, hatchling, and post‐fledging. We additionally modeled the effect of NDVI during the overwintering period in year *t* − 1 to investigate a carry‐over effect of wintering ground condition on probability of survival to the subsequent year.

#### Estimation of fecundity

Fecundity was decomposed into clutch size and the probability that an egg produced a fledgling. Fecundity is known to differ between ages in these populations, with evidence for early‐life improvement and late‐life decline in clutch size and probability of nest success (Fay et al., 2021). Therefore, we separated nest data according to the stage class of the breeding female: 1‐year‐old local recruits (female banded as a juvenile at either site), immigrants (unbanded female detected for the first time in year *t*, presumably a 1‐year‐old disperser into either population), and adults (banded female 2 years old and older). We summarized reproductive data at the population level for the two sites separately (i.e., number of broods [$b_{a,s,t}$], clutch size [$c_{a,s,t}$], and number of birds fledged [$f_{a,s,t}$]). Estimated fecundity parameters ($\kappa_{a,s,t},\zeta_{a,s,t}$) varied between stage classes *a*, sites *s*, and years *t*.

##### Estimation of clutch size

The total observed clutch size at the population level ($c_{a,s,t}$; the total number of eggs in all nests) per stage class, site, and year was modeled as a Poisson random variable where:

(14) $$c_{a,s,t}˜\text{Poisson}\left(\kappa_{a,s,t}\times b_{a,s,t}\right).$$

$b_{a,s,t}$ was the total number of broods (i.e., initiated nests, inclusive of failed nests) across all nest boxes per stage class *a*, site *s*, and year *t*, and $\kappa_{a,s,t}$ was the average estimated clutch size across all broods of females of stage *a*, in site *s*, in year *t*. $\kappa_{a,s,t}$ was modeled on the log scale as a function of a stage and site‐specific clutch size mean across all years ($\mu_{\kappa ,a,s}$), with annual variation around the mean ($\varepsilon_{\kappa ,a,s,t}$), where:

(15) $$\log\left(\kappa_{a,s,t}\right)=\mu_{\kappa ,a,s}+\varepsilon_{\kappa ,a,s,t}.$$

(16) $$\varepsilon_{\kappa ,a,s,t}˜\text{normal}\left(0,\;\sigma_{\kappa ,a,s,t}^{2}\right).$$

We did not expect that any annual variation in clutch size would be explained by environmental conditions (Sanz, 2001), and therefore did not model the effects of covariates on $\kappa_{a,s,t}$.

##### Estimation of the probability to fledge

The total number of fledglings at the population level ($f_{a,s,t}$) per stage class, site, and year was modeled as a binomial random variable, where:

(17) $$f_{a,s,t}˜\text{binomial}\left(c_{a,s,t},\zeta_{a,s,t}\right).$$

$c_{a,s,t}$ was the total observed clutch size at the population level per stage class, site, and year, and $\zeta_{a,s,t}$ was the estimated probability of fledging (i.e., probability that each individual egg survived, hatched, and fledged). Exploratory analysis showed that the probability of fledging $\left(\zeta_{a,s,t}\right)$ at each site had some temporal correlation between the stage classes (Appendix S8). We explicitly accounted for and estimated this correlation among stage classes using correlated random effects and modeled the variance–covariance matrix using Cholesky decomposition with parameter expansion, following the approach of Chen and Dunson (2003) and Fay et al. (2021, 2022); Appendix S9. This approach allowed us to model year effects on probability of fledging while accounting for potential covariance between the stage classes in a computationally efficient manner. Ignoring such correlation structures can lead to biased estimates of shared temporal variance, particularly in integrated models where parameters are estimated jointly from multiple data sources. We accounted for among‐year variation in probability of fledging using mean, minimum, and maximum daily temperature averages and cumulative precipitation for the time periods corresponding to nest initiation/incubation and hatchling growth. We also modeled the effect of NDVI during the overwintering period in year *t* − 1 to investigate a carry‐over effect of wintering ground condition. To investigate the effect of dormice on probability of fledging chicks, we modeled an effect of mast years in year *t* and a time‐lagged effect of mast in years *t* − 1. Finally, we modeled the effect of nest initiation date, as there was a notable decrease in nest initiation date for all stage classes in both sites (Appendix S3). Like with apparent survival, environmental covariates were included as covariate effects using a generalized linear model (Appendix S9).

#### Estimation of immigrants

The numbers of immigrants of sex *j* ($N_{j,\mathrm{im},s,t}$) were estimated without explicit data, given that there was not complete capture in our system. We specified the number of immigrants as:

(18) $$N_{j,\mathrm{im},s,t}˜\text{Poisson}\left(\omega_{j,s}\right),$$

where $\omega_{j,s}$ was the expected numbers of newly immigrated individuals of sex *j* at site *s* in each year excluding the first year of the monitoring. Immigrants were assumed to be 1‐year‐old natal dispersers. $\omega_{j,s}$ was log‐normally distributed but without temporal variation because we did not have any interest in testing covariates against hypotheses related to immigration. Although there was no temporal variability in the parameter estimation itself, there was still temporal variability in the realized number of immigrants due to demographic stochasticity from the Poisson distribution.

#### Estimation of population sizes

We modeled the number of individuals for each sex *j*, site *s*, and year *t* $\left(N_{j,s,t}\right)$ using a Gaussian state space model (SSM; De Valpine & Hastings, 2002) based on the counts of next boxes occupied annually ($y_{j,s,t}$). The Gaussian SSM decomposed the variance in the observed counts of nest boxes into site‐specific residual error ($\tau_{y,s}$) from the underlying true dynamics of the states $N_{j,s,t}$ (De Valpine & Hastings, 2002). The counts were normally distributed as:

(19) $$y_{j,s,t}˜\log\;\text{normal}\left(N_{j,s,t},\tau_{y,s}\right).$$

#### Model sets and stepwise covariate selection

We analyzed eleven models from our set of IPMs (Appendix S10), where covariates were tested independently for both survival and fecundity submodels. Any covariate effect (β) with 85% Bayesian credible intervals (CRI) that did not overlap zero from their respective, separate IPMs were combined in the final model and used for the variance decomposition analysis. We used 85% CRIs to assess covariate effects during model selection to avoid prematurely excluding covariates with moderately strong signals. This threshold strikes a balance between Type I and Type II errors by reducing the risk of excluding covariates with real biological relevance (Type II error), while maintaining some protection against including false positives (Type I error). In this first step of the model selection process, using 95% CRIs may be overly conservative (Arnold, 2010).

#### Estimating contributions of demographic parameters to population growth rate and decomposition of variance

We used the derived quantities of the temporally varying demographic rates from the fitted IPMs to conduct a retrospective transient life‐table response experiment to estimate the contribution of variation in each demographic component to variation in the realized annual population growth rate (Knape et al., 2023; Koons et al., 2016; Schaub & Kéry, 2022). Following the methods of Knape et al. (2023), we further partitioned the contributions from the different demographic components into contributions from the temporally varying environmental factors and unexplained random variation that affected each demographic component, ultimately linking these contributions back to variation in realized annual population growth rates (see Knape et al., 2023 and Appendix S9: Section S2). We calculated the annual sensitivities of population growth rate to each demographic rate using the analytical derivatives of the population projection equation at each time step, following Knape et al. (2023). We report these sensitivities and contributions to contextualize which covariate effects identified in the IPM were most likely to influence population dynamics. We only investigated female demographic rates in this analysis because all reproductive parameters were modeled as a function of female stage and did not consider male demography.

#### Running the models

To fit the models, we employed Markov Chain Monte Carlo (MCMC) methods within a Bayesian framework using JAGS (Brooks et al., 2004; Plummer, 2012) accessed through R version 4.4.0 (R Core Team, 2021) and package jagsUI (Kellner, 2024). We ran three MCMC chains of 100,000 iterations for each model, discarded the first 10,000 iterations and retained every fifth saved iteration (Schaub & Fletcher, 2015). We specified vaguely informative priors following Kéry and Schaub (2012); Appendix S9. Additional details relating to priors are provided in Appendix S9. We used the Brooks and Gelman diagnostic to assess the convergence of the MCMC simulations (Brooks & Gelman, 1998), which were all below 1.3 and assessed base model fit by posterior predictive *p*‐values (Appendix S11).

## RESULTS

**FIGURE 2:**
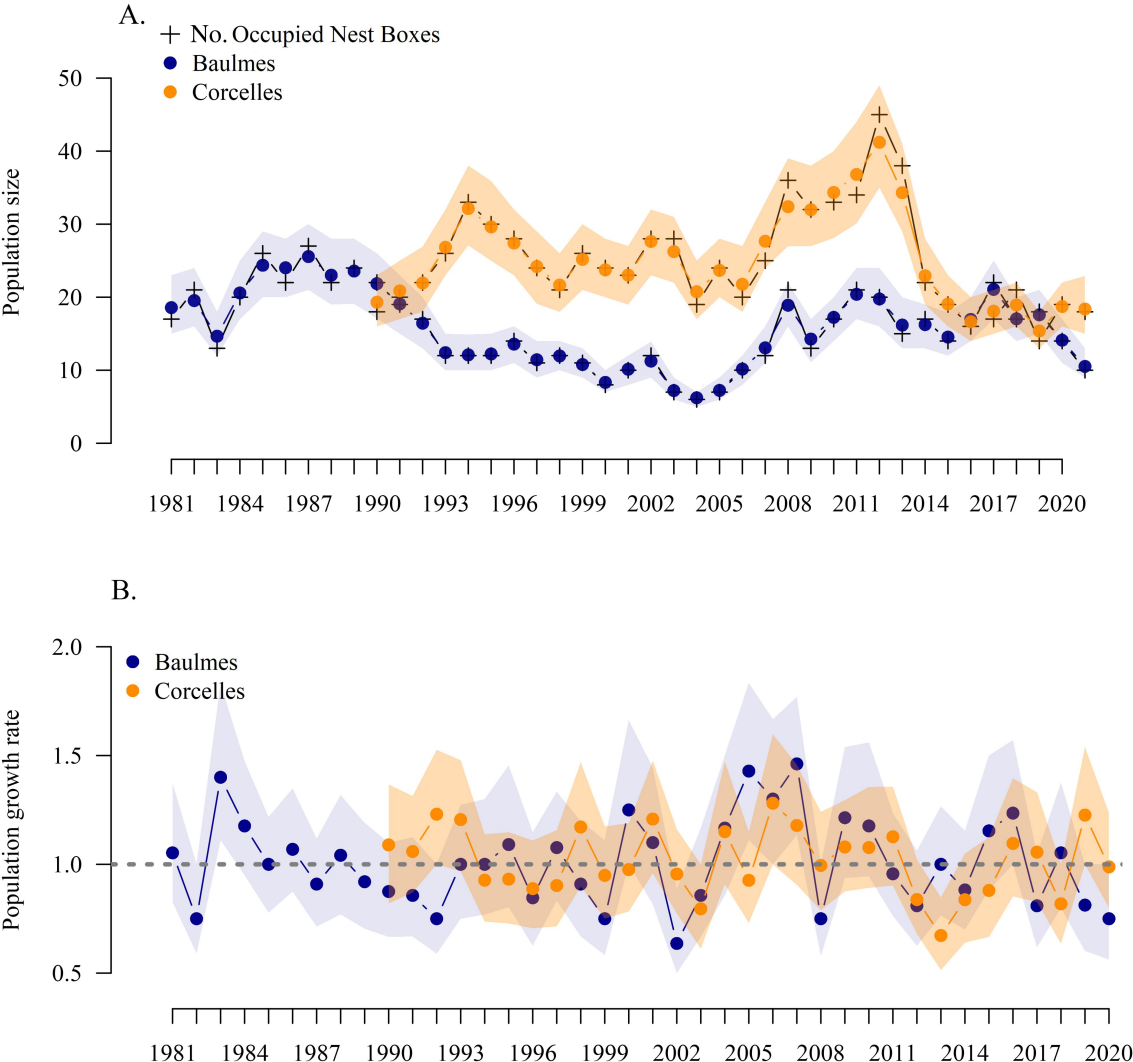
Estimated population sizes (A) and population growth rates (B) of female pied flycatchers in Baulmes and Corcelles from 1980 to 2020. The population sizes are shown with 95% credible intervals shaded in light orange for Corcelles, and in dark blue for Baulmes. The black crosses represent the count of occupied nest boxes at each site. (B) Population growth rates (λ) over time for Baulmes, in blue, and Corcelles, in orange with 95% credible intervals shown from 1981 to 2020. Population growth rates were calculated from the total population abundance of females. The dotted gray line in panel (B) shows the growth of a stable population (λ = 1).

On average, 14.9% and 14.6% of nest boxes were occupied in Baulmes and Corcelles, respectively (Appendix S2), with an average of 25 breeding pairs in Corcelles and 16 breeding pairs in Baulmes (Figure 2). Across all years, both populations experienced a slight decrease in size (Baulmes β = −0.14 [95% CI = −0.26 to −0.02], Corcelles β = −0.12 [95% CI = −0.32 to 0.08]). The years from 2004 until 2012 saw an increase in Baulmes (β = 1.37 [95% CI = 0.93–1.81]) and Corcelles (β = 1.57 [95% CI = 0.93–2.21]; Figure 2A), and both experienced their largest growth rates during this period (Figure 2B). Both populations have declined since 2012 (Baulmes β = −0.53 [95% CI = −1.05 to −0.01], Corcelles β = −1.16 [95% CI = −2.10 to −0.22]).

**FIGURE 3:**
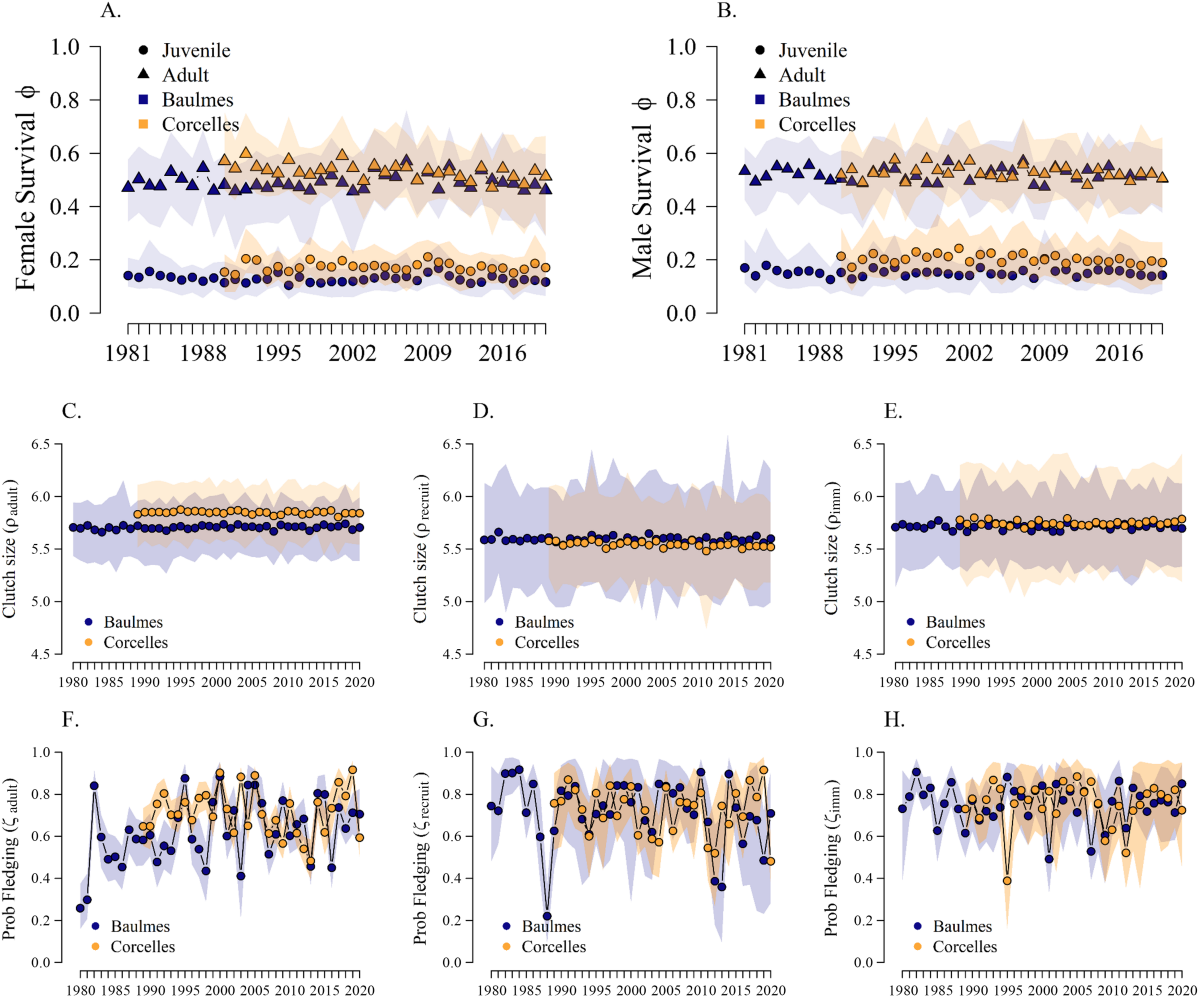
Estimated parameter values taken from the base model without environmental covariates (Model #1, Table 1). (A) Apparent female survival, (B) apparent male survival, (C–E) clutch size, (F–H) probability of fledging. Symbols show posterior means for Baulmes (in blue) and Corcelles (in orange). The shaded areas show the 95% credible intervals.

Adult apparent survival was higher than juvenile apparent survival for males and females in the two populations (Table 1, Figure 3A,B). Male juvenile and adult apparent survival in Corcelles tended to be higher than in Baulmes (Table 1, Figure 3). Clutch size showed remarkably little variability across years and between the two populations (Table 1, Figure 3C–E). Probability of fledging was highly variable across time and was not significantly different between the three age stages (Table 1, Figure 3F–H). The estimated numbers of immigrants were generally low and were higher in females than in males (Table 1; Appendix S12).

**TABLE 1:** Posterior means and 95% credible intervals (designated in parentheses) of mean (over time) demographic parameters from the base model excluding environmental covariates.

Name	Baulmes	Corcelles
Population growth rate	0.98 (0.96–1.00)	0.99 (0.97–1.01)
Female juvenile survival	0.13 (0.07–0.21)	0.17 (0.10–0.26)
Female adult survival	0.49 (0.35–0.64)	0.54 (0.39–0.68)
Male juvenile survival	0.15 (0.08–0.24)	0.20 (0.12–0.31)
Male adult survival	0.52 (0.37–0.67)	0.53 (0.37–0.68)
Clutch size—Recruits	5.62 (5.02–6.25)	5.59 (5.05–6.12)
Clutch size—Immigrants	5.70 (5.26–6.19)	5.79 (5.23–6.42)
Clutch size—Adults	5.69 (5.39–6.01)	5.85 (5.58–6.16)
Probability of fledging—Recruits	0.72 (0.22–0.96)	0.72 (0.44–0.93)
Probability of fledging—Immigrants	0.76 (0.49–0.93)	0.76 (0.44–0.95)
Probability of fledging—Adults	0.62 (0.28–0.90)	0.72 (0.49–0.92)
No. female immigrants	3.72 (2.45–4.93)	2.57 (0.57–4.78)
No. male immigrants	2.41 (0.90–3.95)	1.16 (0.12–3.35)

*Note*: The population growth rate mean was calculated as the geometric mean.

**FIGURE 4:**
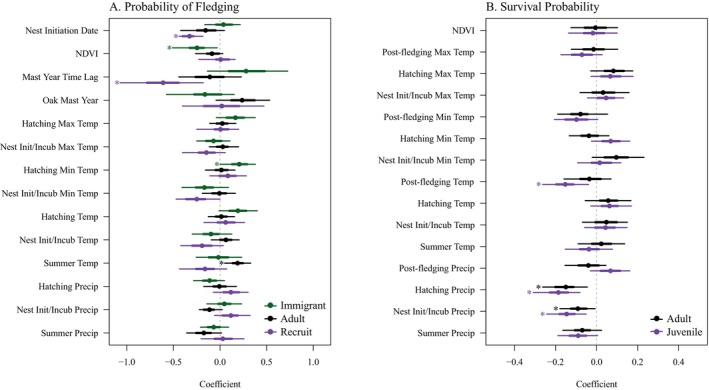
Estimated slopes for all covariate effects on the probability of fledging (A) and of survival (B) for covariates tested in individual integrated population models (IPMs). Tested covariates are on the *y*‐axis and coefficient estimates are on the *x*‐axis. The error bars show 50% (thick) and 85% (thin) credible intervals. Estimates that had an 85% credible interval that did not overlap zero are denoted with an asterisk (*) and were used in the variance decomposition analysis. NDVI, Normalized Difference Vegetation Index.

Environmental and phenological covariates influenced demographic rates in our single‐covariate IPMs. Probability of fledging from nests of female recruits experienced negative effects of nest initiation dates (β = −0.33, 95% CRI = [−0.45 to −0.17]; Figure 4A) and the year following a mast year (i.e., time‐lagged effect; β = −0.61, 95% CRI = [−1.14 to −0.04]). Probability of fledging from nests of immigrants was negatively influenced by overwintering NDVI (β = −0.37, 95% CRI = [−0.81 to 0.02]; Figure 4A) and positively influenced by the hatchling‐period minimum temperature (β = 0.21, 95% CRI = [0.00–0.46]; Figure 4A). Probability of fledging from adult nests was positively influenced by overall summer temperatures (β = 0.19, 95% CRI = [0.01–0.36]; Figure 4A).

Precipitation influenced apparent survival of juveniles and adults (Figure 4B). Higher cumulative precipitation during the nest initiation/incubation period and the hatchling period resulted in lower juvenile (NII β = −0.15, 95% CRI = [−0.27 to −0.03], H β = −0.19, 95% CRI = [−0.32 to −0.05]) and adult (NII β = −0.09, 95% CRI = [−0.20 to 0.01], H β = −0.15, 95% CRI = [−0.29 to −0.02]) apparent survival (Figure 4B). For juveniles, higher temperatures during the post‐fledging period (β = −0.15, 95% CRI = [−0.28 to 0.01]) resulted in lower survival (Figure 4B).

From these individual covariate models, we constructed the IPM that was used for the variance decomposition analysis. For survival, we included the covariates nest initiation/incubation period precipitation, hatchling period precipitation, and post‐fledging average temperature and for the probability of fledging we included nest initiation date, overwintering NDVI, mast year time lag, hatchling period minimum temperature, and summer average temperature (Appendix S10).

**FIGURE 5:**
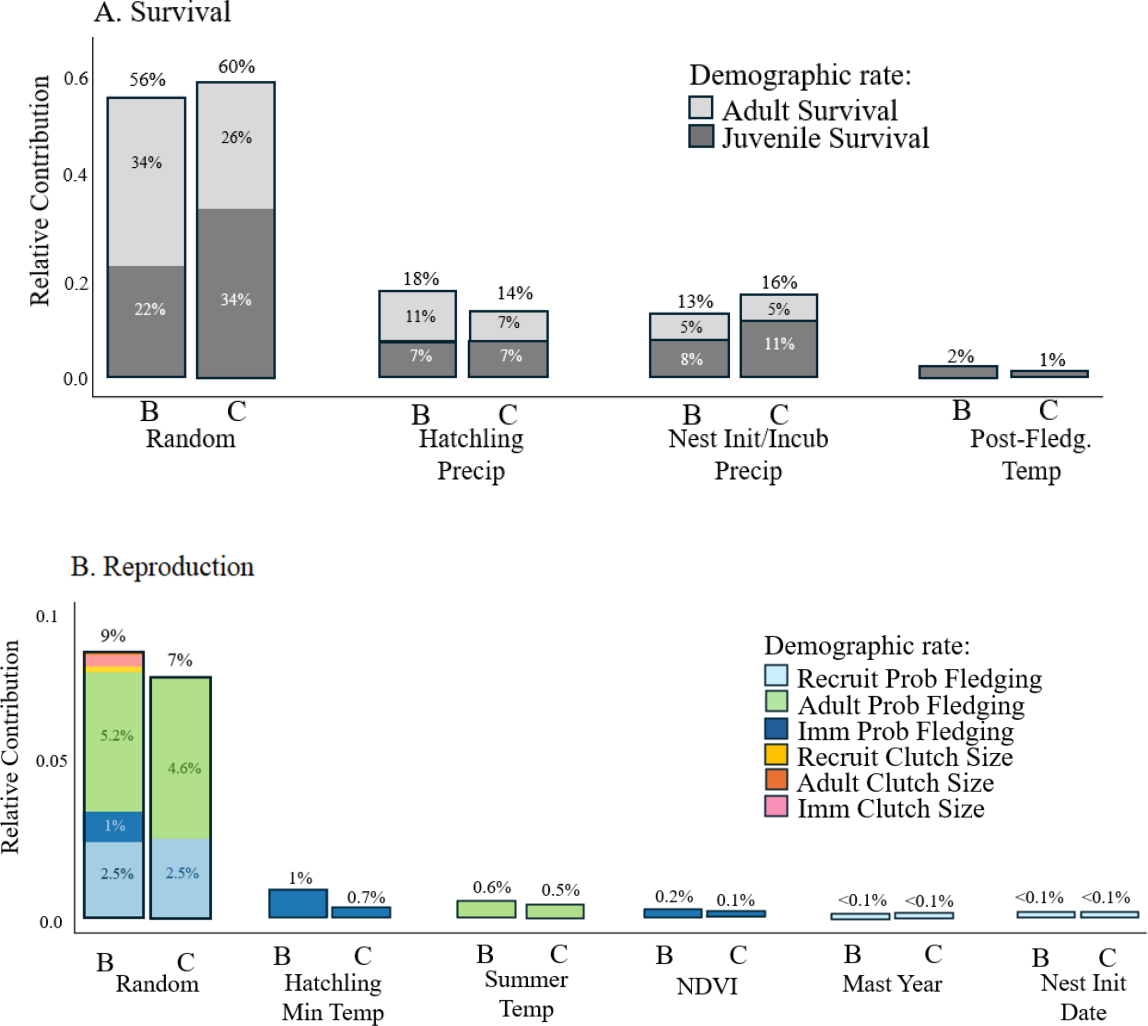
Relative contributions of covariates to variation in the realized population growth rate in Corcelles (“C”) and Baulmes (“B”) for demographic rates and associated tested covariates related to survival (A) and fecundity (B). The relative contribution to variation in the realized population growth rates is separated along the *x*‐axis by contributing covariate, and the colors indicate through which demographic rate the covariate is acting (“Demographic rate”). Cumulative contributions per site (C, B) per covariate are shown as a percentage of variation explained. Percentages shown are the relative contributions of individual covariates. When multiple covariates contributed to one demographic rate, percentages are listed for all covariates contributing >1%. Calculated from the model including all covariates which did not have an 85% Bayesian credible interval overlapping 0 from the single covariate integrated population model (IPM) analysis (Model #12, Appendix S10).

Decomposition of the variance in the realized population growth rate into contributions from vital rates revealed that most variation was driven by apparent survival (89% in Baulmes, 91% in Corcelles), with minor contributions from fecundity (11% in Baulmes, 9% in Corcelles; Figure 5). Random unaccounted for variation in apparent juvenile survival accounted for 22.1% (95% CRI = 0.02%–54.3%) of variation in population growth rate in Baulmes and 34.0% (95% CRI = 1.2%–71.5%) of variation in population growth rate in Corcelles, while random unaccounted for variation in apparent adult survival accounted for 33.6% (95% CRI = 0%–68.4%) of variation in population growth rate in Baulmes, and 26.1% (95% CRI = 0%–60.1%) of variation in population growth rate in Corcelles. Random unaccounted for variation in fecundity accounted for 9% and 7% of variation in population growth rates in Baulmes and Corcelles, respectively (cumulative sums of recruit, adult, and immigrant probability of fledging and clutch size random relative contributions; Figure 5B).

Annual sensitivities of population growth rate to each demographic rate indicated that population growth rate was most sensitive to variation in juvenile and adult survival, while sensitivities to reproductive parameters were close to zero (Table 2). Population growth rate was most sensitive to changes in juvenile and adult survival, which together accounted for roughly 90% of the total sensitivity across both populations (Table 2). These results indicate that fluctuations in survival, particularly juvenile survival, play a large role in driving interannual changes in population growth rates compared to variation in reproductive parameters. This suggests that environmental or ecological drivers influencing survival are likely to have the greatest impact on population dynamics in this system.

**TABLE 2:** Relative sensitivities of population growth rate (λ) to each demographic parameter, averaged across years.

Name	Baulmes	Corcelles
Female juvenile survival	0.392	0.532
Female adult survival	0.504	0.380
Clutch size—Recruits	0.002	<0.001
Clutch size—Immigrants	0.003	<0.001
Clutch size—Adults	0.001	<0.001
Probability of fledging—Recruits	0.025	0.026
Probability of fledging—Immigrants	0.022	0.003
Probability of fledging—Adults	0.051	0.058

*Note*: Values are normalized to sum to 1 within each population. These values indicate the influence of each rate on population growth rate, independent of their temporal variability. Contributions are shown separately for Baulmes and Corcelles.

In both populations, precipitation influenced apparent juvenile survival and contributed the most variation of any environmental covariate to variation in population growth rate (15.2% in Baulmes, 18.4% in Corcelles; cumulative sums of the relative contribution from nest initiation/incubation and hatchling precipitation; Figure 5A). When broken down into specific periods of the breeding season, the carryover effect of cumulative precipitation acted negatively on apparent juvenile survival during nest initiation/incubation and explained the most variation at each site (Baulmes: 8.4% [95% CRI = 0%–22.7%]; Corcelles: 11.1% [95% CRI = 0%–32.7%]), followed by cumulative precipitation during the hatchling period (Baulmes: 7.4% [95% CRI = 0%–20.5%]; Corcelles: 7.1% [95% CRI = 0%–24.5%]). Cumulative precipitation also acted negatively through apparent adult survival during the nest initiation/incubation period (Baulmes: 5.3% [95% CRI = 0%–19.6%]; Corcelles: 5.4% [95% CRI = 0%–18.2%]) and hatchling period (Baulmes: 11.2% [95% CRI = 0%–28.4%]; Corcelles: 7.0% [95% CRI = 0%–21.1%]).

Environmental covariates had minor influences on population growth rate when acting through fecundity (Figure 5B). The largest contribution to the variation in population growth rate came from unexplained random noise. Hatchling period minimum temperature, summer temperature, NDVI, mast year, and nest initiation dates each contributed ≤1% to the variation in population growth rates (Figure 5B).

## DISCUSSION

We identified complete demographic pathways by linking environmental covariates to demographic rates and their consequences on population dynamics to determine which covariates and time periods had the largest impact on population dynamics. We showed empirically that linking environmental covariates to demographic rates does not sufficiently explain or predict population‐level consequences, and that decomposing variation along the complete demographic pathway is a necessary step to appropriately identify how covariates influence population dynamics. The tested covariates did not explain as much variation in population growth rate as random variation, which could have been mistakenly attributed to covariates without variance decomposition. Such a mechanistic understanding is particularly important for predicting future population changes in the face of changing environmental conditions.

Cumulative precipitation during key periods of the breeding season significantly affected juvenile and adult survival in both studied pied flycatcher populations, while nest initiation date, NDVI during nonbreeding, mast years, and temperatures during breeding influenced probability of fledging. Since variation in population growth rates was primarily driven by variation in juvenile and adult apparent survival, precipitation during breeding had the strongest effect on population growth rates by negatively influencing juvenile survival. While several environmental factors significantly impacted the probability of fledging, their overall influence on population growth was minimal, showing that environmental factors may have significant effects on individual or demographic processes, but that they could ultimately have little impact on population growth rates. Our findings reveal that increased precipitation during the nest initiation/incubation and hatchling stages had negative carry‐over effects on juvenile survival during the post‐fledging and overwintering periods. This finding adds to the growing body of work highlighting how carry‐over effects during the breeding season significantly impact subsequent juvenile survival (e.g., Dawson et al., 2005; Harrison et al., 2011). Moreover, increased precipitation reduced adult apparent survival, likely due to the increased energetic demands of caring for eggs and hatchlings in challenging conditions and reduced availability of aerial prey.

Our results indicate that fluctuations in survival, particularly juvenile survival, are a major driver of interannual changes in population growth compared to variation in reproductive parameters. This pattern reflects a combination of high temporal variability in survival and relatively high sensitivity of population growth rate to changes in survival rates, especially during the juvenile stage. In contrast, fecundity parameters, though occasionally variable, had lower sensitivities and weaker associations with annual changes in population growth. We found limited evidence of strong covariation among vital rates, suggesting that the effects of individual parameters on population growth were not substantially offset by compensatory dynamics. As described by Koons et al. (2017), the contribution of a demographic rate to the variance in population growth can be low due to low temporal variability, low sensitivity, or negative covariation with other vital rates; our results suggest that both sensitivity and variability in survival were sufficiently high to dominate temporal variation in population growth.

Our estimates of abundance were derived from counts of occupied nest boxes. Key model assumptions were that nonbreeders were not playing a large role in population dynamics and that we detected all breeding pairs at each site each year. Experimental removal of pied flycatchers has shown complete replacement by younger males and older females, indicating presence of nonbreeding surpluses (Both et al., 2017). It is possible that there were nonbreeding surpluses buffering the two populations, but given the low occupancy of nest boxes, we expect that nonbreeding surpluses were minimal. It is also possible that there could have been some individuals breeding in natural cavities that went undetected, but the extent to which this occurred is unknown and suspected to be low. Densities of pied flycatcher populations are normally not limited by space, but by the availability of nest sites (von Haartman, 1972). Given the average nest box occupancy at Baulmes and Corcelles was low and the well‐documented preferences for nest boxes over natural cavities, it is likely that few, if any, natural cavities were used instead of nest boxes (Lundberg & Alatalo, 2010).

We estimated relatively stable and high apparent survival probabilities in juveniles and adult females and males. Overall, apparent adult flycatcher survival probabilities were consistent with other studies of similar passerines (adult survival range: 0.5–0.6) and are among the highest reported pied flycatcher survival probabilities documented to date (Ravussin et al., 2007). While we could not distinguish mortality from any dispersal events outside of our study populations, pied flycatchers have a low natal dispersal distance (Chernetsov et al., 2006) and high site fidelity in adults, which supports the conclusion that our findings on juvenile and adult apparent survival closely reflect true survival dynamics (Lundberg & Alatalo, 2010).

For both juveniles and adults, higher precipitation during nest initiation/incubation and hatchling periods lowered apparent survival. In the nest initiation/incubation and hatchling life cycle stages, juveniles are fully reliant upon parental care for feeding and thermoregulation. Increases in precipitation can cause a decrease in food availability because invertebrates are less active (Avery & Krebs, 1984) or an increased cost to thermoregulation (Veistola et al., 1997). A lower amount of food delivered to nestlings due to increased precipitation can result in a decline in fledgling body condition and nestling growth, which has been shown to strongly affect juvenile survival (Siikamäki, 1996). Bad weather conditions also increase the amount of energy needed for adult birds to compensate for changes in prey activity (Avery & Krebs, 1984), alter foraging patterns (Veistola et al., 1997), or to meet increased energy demands of hatchlings (Järvinen & Ylimaunu, 1986), ultimately having a negative consequence on their survival as poor body condition before migration can decrease survival chances (Seward et al., 2013).

Overall, precipitation was the most important covariate in explaining environmental‐driven variation in population growth rate in Baulmes and Corcelles during the nest initiation/incubation and hatchling periods. Across the periods of the breeding season, precipitation acting on juvenile survival during the nest initiation/incubation and hatchling periods played a larger role in driving variation in population growth rate than precipitation acting on adult survival in both populations. The large consequences of increased precipitation on survival provide some evidence for a “silver‐spoon” effect in pied flycatchers, where better early life conditions in young confer increased probability of survival that could continue into later life stages or adulthood (Lindström, 1999). In populations of pied flycatchers experiencing declines, conservation actions that prioritize the preservation of habitats with both high insect abundance and reliable prey availability (i.e., high habitat quality) could best augment juvenile and adult survival. Such habitats may buffer the effects of unfavorable environmental conditions, particularly increased precipitation during nest initiation, incubation, and hatchling periods, by ensuring sufficient food availability for foraging adults. Given that precipitation can suppress insect activity even in areas with high biomass, both the quantity and accessibility of prey are critical. However, projecting future rainfall patterns at spatial and temporal scales relevant to prey availability remains a challenge, which limits the precision with which such high‐quality habitats can currently be identified.

Environmental covariates such as nest initiation date, overwinter NDVI, mast years, and temperatures significantly impacted the probability of fledging in nests laid by recruits, immigrants, and adults. Despite having significant effects on the probability of fledging, when translated into consequences on population growth rates, these environmental covariates acting on the probability of fledging contributed very little to variation in population dynamics (1.9% and 1.4% in Baulmes and Corcelles, respectively). These results show that covariates may have significant effects on parameter estimates themselves, but this could ultimately have few consequences for population growth rates. It is possible that the effect on population growth may be buffered or diluted by other demographic processes such as compensatory dynamics or lability. These findings underscore the need to consider both the strength of covariate effects on demographic rates and their relative contribution to overall population dynamics when interpreting ecological significance. We provide further commentary on interesting, but ultimately not influential, relationships between covariates and the probability of fledging in Appendix S13.

Over half the variation in our population growth rates remained unexplained by the environmental and phenological covariates we selected, suggesting that other important drivers of demographic variation were not captured. While we focused on key environmental variables during breeding and nonbreeding periods, other biologically relevant covariates, like habitat pollutant concentrations (Eeva et al., 2002), likely play a significant role in driving survival and fecundity and merit further testing. Landscape‐level changes such as habitat fragmentation, resource availability, and predator abundance may interact with environmental covariates to influence demographic rates in complex ways. Other components such as individual heterogeneity that can arise from age, experience, physiology, or behavior may not be captured by population‐level environmental covariates. Critically, our model only included key environmental covariates during two periods of the migratory birds' lifecycle, the breeding and nonbreeding periods. Environmental conditions during migration, which may substantially influence population growth, were unaccounted for due to data limitations. Future studies should incorporate data on conditions on migration routes as it becomes available.

Our results closely parallel those of Knape et al. (2023), who found that environmental covariates explained ~47% of the variation in population growth rates in their study. Similarly, in our analysis, a substantial portion of the variation remained unexplained, with environmental covariates explaining ~35% and ~33% of the variation in population growth rates of Baulmes and Corcelles, respectively. This reinforces the conclusion that while certain covariates can significantly influence individual demographic rates, their aggregate impact on population‐level outcomes may be modest. Both studies underscore the complexity of demographic systems, where multiple, potentially interacting processes, including stochastic events and unmeasured biotic interactions, contribute to population dynamics. Including more high‐quality biologically relevant environmental covariates in future models could help clarify more of this unexplained variation. We also note that consideration of the magnitude of the variance in population growth rates is critical when interpreting the ecological significance of the covariate effects. In systems where variance in population growth is large, even a modest proportion of explained variation may reflect substantial demographic sensitivity to environmental covariates (Knape et al., 2023). At least in smaller populations, such as in the studied pied flycatcher populations, we cannot expect to find 100% explanation through covariates, as other mechanisms such as demographic stochasticity or density dependence can also impose temporal variation in demographic rates.

A key strength of our analysis was the use of a long‐term, individual‐based dataset that enabled us to estimate fully time‐varying survival and fecundity parameters within an IPM framework (Schaub & Kéry, 2022). A long time series was necessary for assessing the influence of environmental covariates on demographic rates and decomposing their contributions to variation in realized population growth (Knape et al., 2023). However, we recognize that implementing a similar approach in other systems may be constrained by data availability. Specifically, reliable estimation of time‐varying demographic parameters requires consistent annual data on marked individuals for survival estimation, robust measures of nest success parameters, and ideally, information on immigration or recruitment. The variance decomposition approach requires sufficient temporal variation in both demographic rates and covariates to yield meaningful results (Koons et al., 2016). Even when these conditions are met, fitting fully time‐varying models can be computationally intensive and may present challenges in convergence or identifiability (Newman et al., 2023). For systems with more limited data, simplifications such as pooling across years or reducing model complexity may be necessary at the expense of reduced inference. We encourage future studies to consider these trade‐offs and to tailor the level of model complexity to the structure and quality of available data while striving to retain biological realism (Newman et al., 2023).

There were clear negative effects of increased precipitation during the nest initiation/incubation and hatchling stages on juvenile ability to survive the post‐fledging and overwinter periods and for adult apparent survival, likely due to higher energy demands for care (Öberg et al., 2015) and reduced aerial prey availability (Avery & Krebs, 1984). Given growing concern over pied flycatcher declines across parts of their range (González‐Braojos et al., 2017; Goodenough et al., 2009), our results offer timely insight: conservation strategies should consider how shifting precipitation patterns, rather than temperature alone, may differentially impact survival and recruitment. More broadly, our study demonstrates the value of integrative, process‐based approaches for informing targeted conservation in the face of climate change. Climate change is altering precipitation patterns in Switzerland (IPCC, 2023), and models predict a decline in cumulative summer precipitation (Zubler et al., 2014), potentially benefiting pied flycatcher populations by increasing juvenile and adult survival due to increased insect availability. The same models are inconclusive about the magnitude and direction of cumulative precipitation in the spring (Zubler et al., 2014). The uncertainty of how precipitation regimes will change during the period of highest susceptibility for juveniles and adult pied flycatchers means that we must exhibit caution when predicting the vulnerability of these populations to climate change.

## AUTHOR CONTRIBUTIONS

All authors conceived the ideas. Ellen C. Martin analyzed the data and led the writing of the manuscript. Thomas V. Riecke and Michael Schaub provided methodological support. Pierre‐Alain Ravussin and Daniel Arrigo collected and managed the data. All authors contributed critically to the drafts and gave final approval for publication.

## CONFLICT OF INTEREST STATEMENT

The authors declare no conflicts of interest.

## Supporting information


Appendix S1.

Appendix S2.

Appendix S3.

Appendix S4.

Appendix S5.

Appendix S6.

Appendix S7.

Appendix S8.

Appendix S9.

Appendix S10.

Appendix S11.

Appendix S12.

Appendix S13.

## Data Availability

Data and code are available on Zenodo as follows: Data (Martin, 2025b), https://doi.org/10.5281/zenodo.17530076; code (Martin, 2025a), https://doi.org/10.5281/zenodo.17529861.
