# Supplementary material for: Identifying the demographic pathways linking environmental covariates to population dynamics in an avian migrant
Source: Ecol Appl. 2026 Jan 5;36(1):e70166. doi: 10.1002/eap.70166 (PMC12770812; doi:10.1002/eap.70166)

Identifying the demographic pathways linking environmental covariates to population dynamics in an avian migrant

Ellen C. Martin, Thomas V. Riecke, Pierre-Alain Ravussin, Daniel Arrigo & Michael Schaub

Ecological Applications

Appendix S1

Figure S1: Location of the two nest box study sites of European pied flycatchers (Ficedula hypoleuca), Baulmes (46°47′N/6°32′ E) and Corcelles (46°50′ N/6°42′ E; A), in the canton of Vaud in Switzerland (B). Baulmes was surveyed from 1980 until 2020, and Corcelles was surveyed from 1989 until 2020.

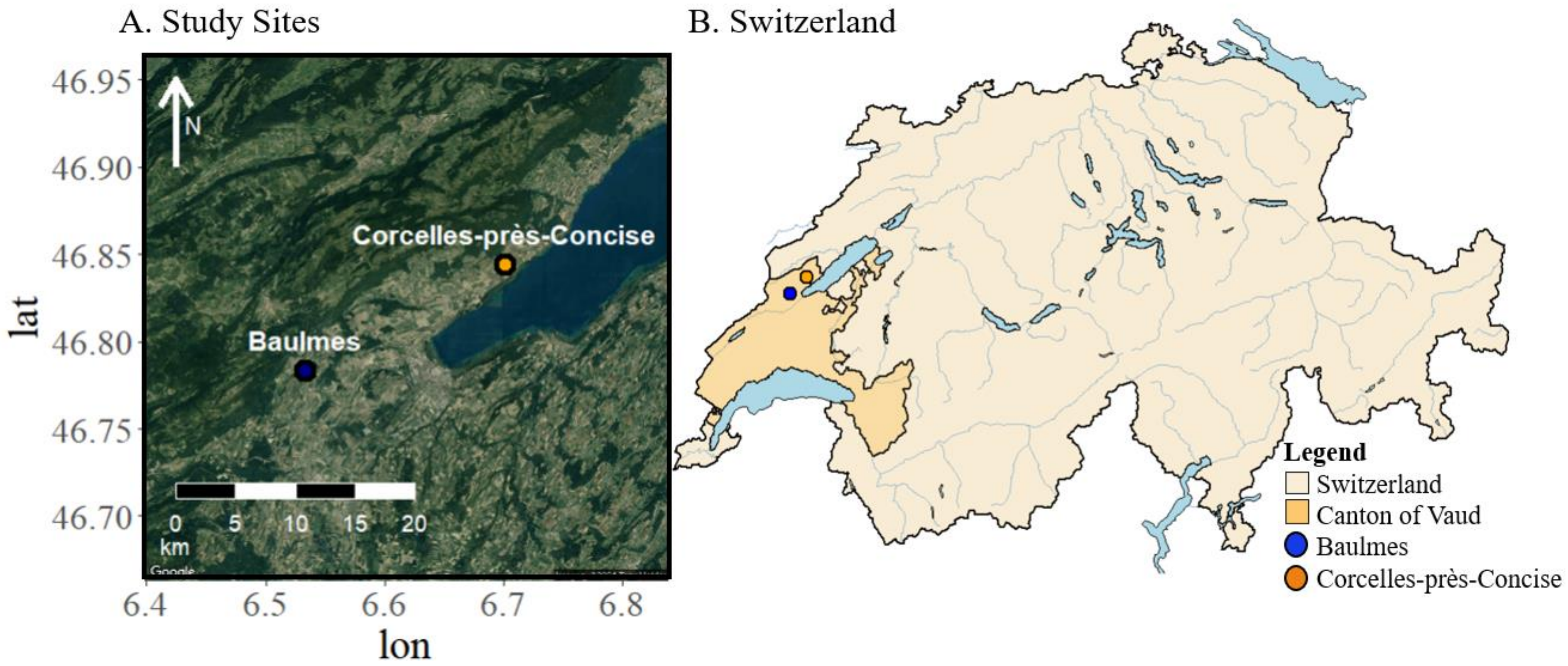

Supplement: Supplementary file 1 — Appendix S1. [file EAP-36-e70166-s011.pdf]
