# Supplementary material for: Identifying the demographic pathways linking environmental covariates to population dynamics in an avian migrant
Source: Ecol Appl. 2026 Jan 5;36(1):e70166. doi: 10.1002/eap.70166 (PMC12770812; doi:10.1002/eap.70166)

Identifying the demographic pathways linking environmental covariates to population dynamics in an avian migrant

Ellen C. Martin, Thomas V. Riecke, Pierre-Alain Ravussin, Daniel Arrigo & Michael Schaub

Ecological Applications

Appendix S2

Figure S1. Number of available nest boxes (solid lines, primary y-axis) and proportion of nest boxes occupied by pied flycatchers (dotted lines, secondary y-axis) at Baulmes (in blue) and Corcelles (in orange) for the duration of the study period (Baulmes: 1980 – 2020, Corcelles: 1989 – 2020).

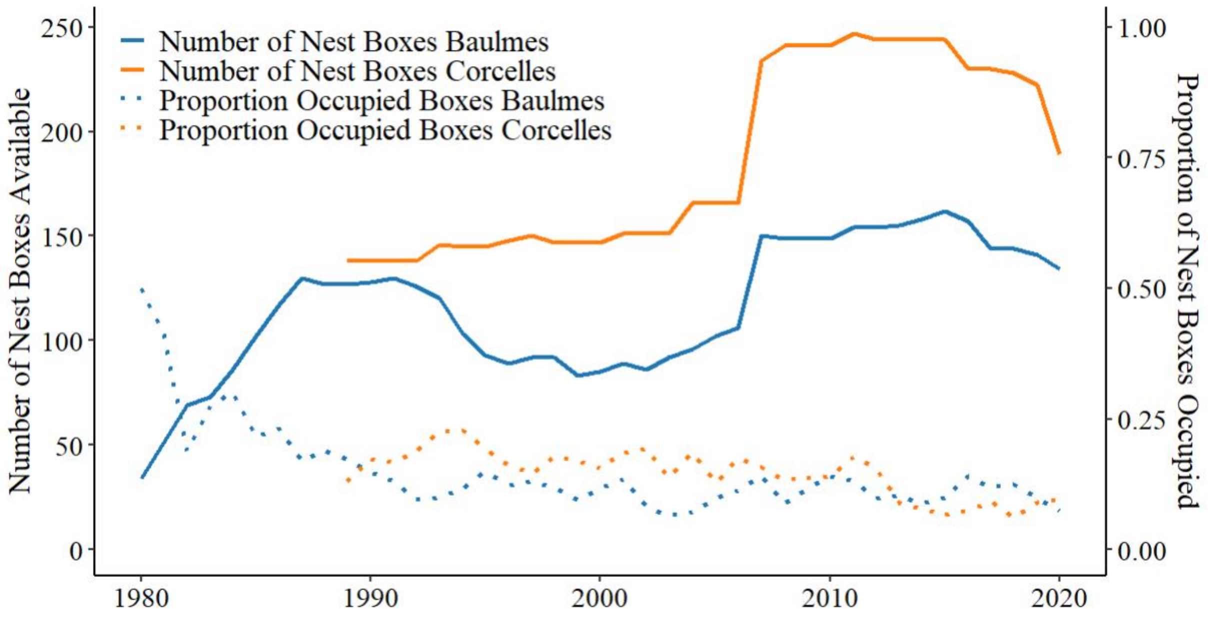

Supplement: Supplementary file 2 — Appendix S2. [file EAP-36-e70166-s006.pdf]
