# Supplementary material for: Identifying the demographic pathways linking environmental covariates to population dynamics in an avian migrant
Source: Ecol Appl. 2026 Jan 5;36(1):e70166. doi: 10.1002/eap.70166 (PMC12770812; doi:10.1002/eap.70166)

Identifying the demographic pathways linking environmental covariates to population dynamics in an avian migrant

Ellen C. Martin, Thomas V. Riecke, Pierre-Alain Ravussin, Daniel Arrigo & Michael Schaub

Ecological Applications

Appendix S3

Figure S1. Mean nest initiation date is shown per year as the ordinal date on the y-axis for recruits (A), adults (B), and immigrants (C) for Baulmes (in blue) and Corcelles (in orange). Mean nest initiation date was defined as the average date that the first egg was laid in all nests per year per population. The trend lines are shown as dotted lines corresponding to the colors of the study sites. Across all years of the study, mean nest initiation date was 137.2 (May 17) for recruits (A), 133.8 (May 14) for adults (B), and 136.4 (May 16) for immigrants (C).

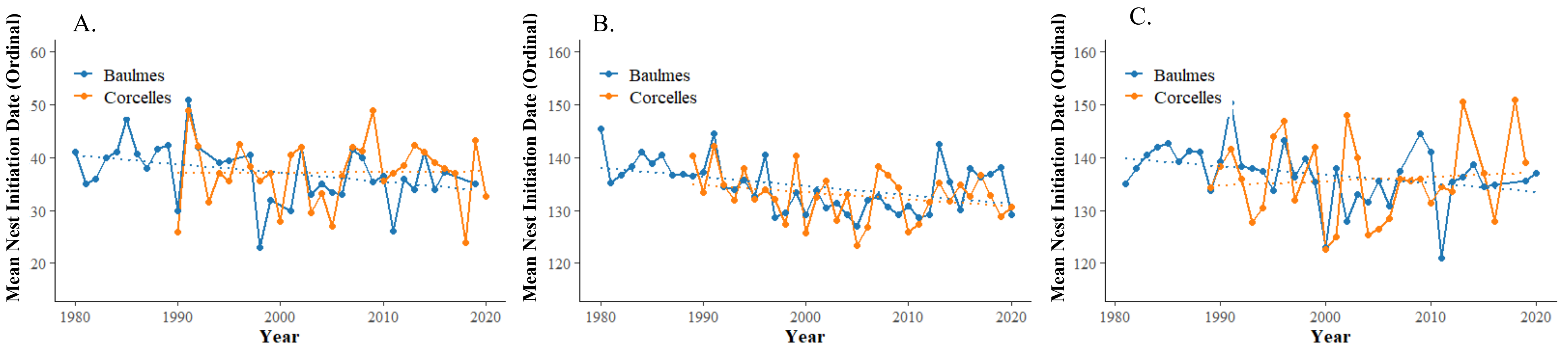

Supplement: Supplementary file 3 — Appendix S3. [file EAP-36-e70166-s012.pdf]
