# Supplementary material for: Identifying the demographic pathways linking environmental covariates to population dynamics in an avian migrant
Source: Ecol Appl. 2026 Jan 5;36(1):e70166. doi: 10.1002/eap.70166 (PMC12770812; doi:10.1002/eap.70166)

Identifying the demographic pathways linking environmental covariates to population dynamics in an avian migrant

Ellen C. Martin, Thomas V. Riecke, Pierre-Alain Ravussin, Daniel Arrigo & Michael Schaub

Ecological Applications

Appendix S4

Figure S1. Annual variation of environmental covariates: mean temperature (“temp”, A, C, E), precipitation (“precip”, B, D, F), mast years (G) for the study sites, and NDVI (H) from the wintering grounds. Plotted values are z standardized annual means (see main text for details). Additional environmental covariates were investigated but not shown due to high correlation with mean temperature were average daily minimum temperatures and average daily maximum temperatures. The dashed black line is the line of best fit, i.e., trend through time.

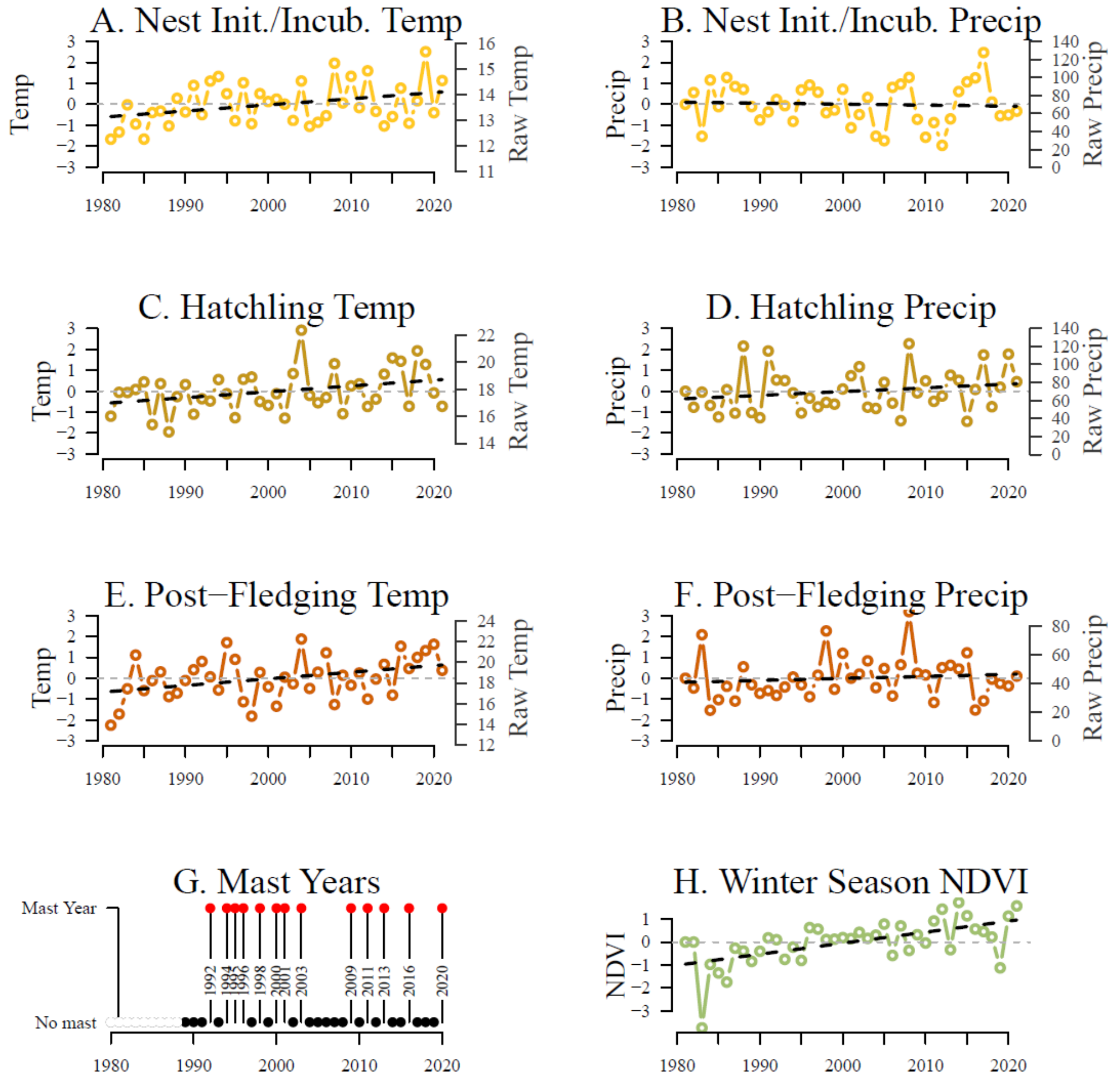

Supplement: Supplementary file 4 — Appendix S4. [file EAP-36-e70166-s005.pdf]
