# Supplementary material for: Identifying the demographic pathways linking environmental covariates to population dynamics in an avian migrant
Source: Ecol Appl. 2026 Jan 5;36(1):e70166. doi: 10.1002/eap.70166 (PMC12770812; doi:10.1002/eap.70166)

Identifying the demographic pathways linking environmental covariates to population dynamics in an avian migrant

Ellen C. Martin, Thomas V. Riecke, Pierre-Alain Ravussin, Daniel Arrigo & Michael Schaub

Ecological Applications

Appendix S5

Figure S1. Correlation matrix of all environmental variables included in survival and probability of fledging analyses. Colors indicate correlation values, from 1 (red) to -1 (blue). Bolded values indicate a correlation > |0.50|.

Covariate definitions:

Imm NID C = nest initiation date immigrants, Corcelles
Ad NID C = nest initiation date adults, Corcelles
Juv NID C = nest initiation date juveniles, Corcelles
Imm NID B = nest initiation date immigrants, Baulmes
Ad NID B = nest initiation date adults, Baulmes
Juv NID B = nest initiation date juveniles, Baulmes
P-F Max Temp = Post-fledging period mean maximum daily temperature
H Max Temp = Hatchling period mean maximum daily temperature
N-I/I Max Temp = Nest initiation/incubation period mean maximum daily temperature
P-F Min Temp = Post-fledging period mean minumum daily temperature
H Min Temp = Hatchling period mean minumum daily temperature
N-I/I Min Temp = Nest initiation/incubation period mean minimum daily temperature
P-F Temp = Post-fledging period mean daily temperature
H Temp = Hatchling period mean daily temperature
N-I/I Temp = Nest initiation/incubation period mean daily temperature
P-F Precip = Post-fledging period cumulative precipitation
H Precip = Hatchling period cumulative precipitation
N-I/I Precip = Nest initiation/incubation period cumulative precipitation

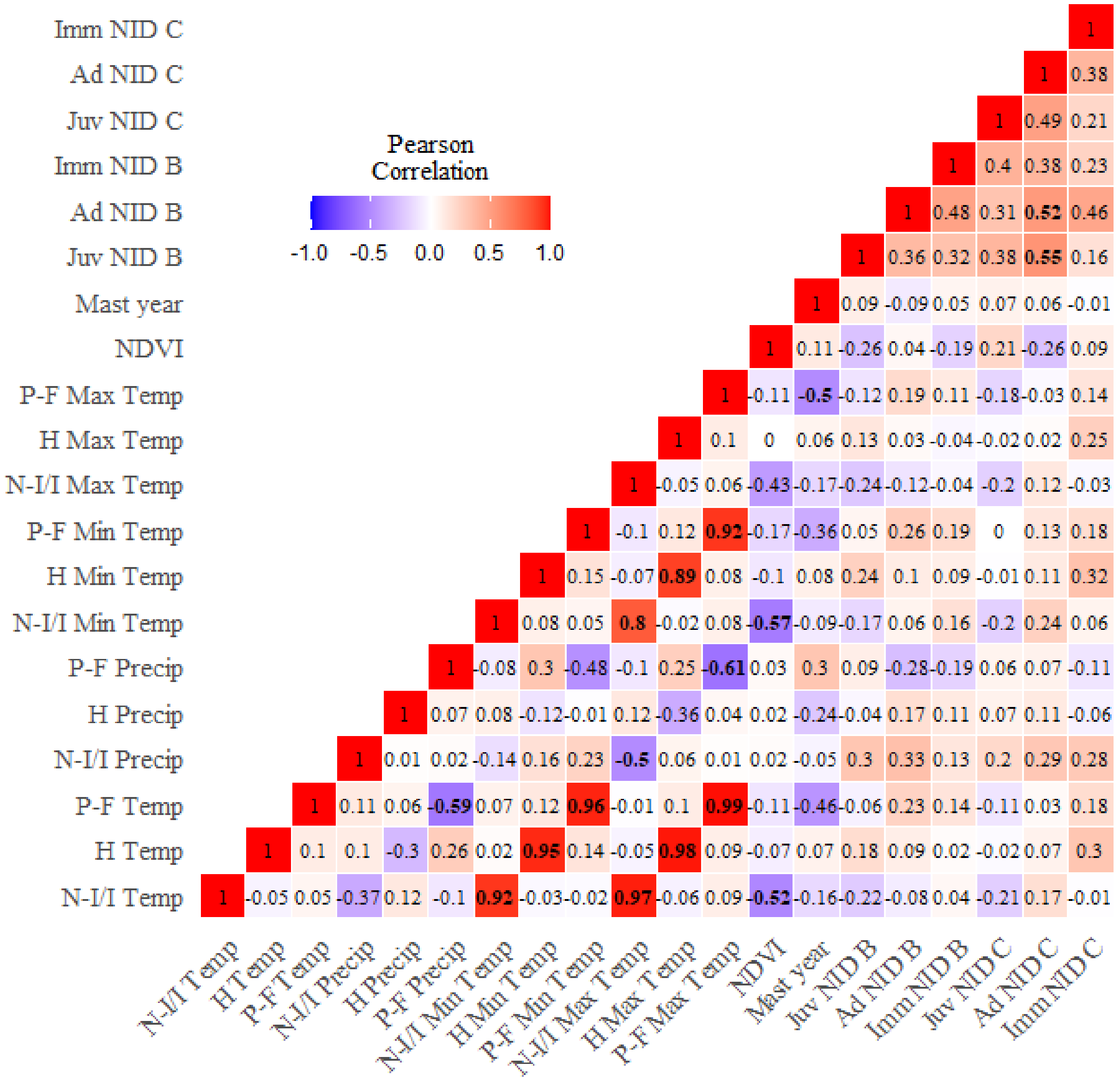

Supplement: Supplementary file 5 — Appendix S5. [file EAP-36-e70166-s010.pdf]
