# Supplementary material for: Identifying the demographic pathways linking environmental covariates to population dynamics in an avian migrant
Source: Ecol Appl. 2026 Jan 5;36(1):e70166. doi: 10.1002/eap.70166 (PMC12770812; doi:10.1002/eap.70166)
Supplement: Supplementary file 6 — Appendix S6. [file EAP-36-e70166-s009.pdf]

Identifying the demographic pathways linking environmental covariates to population dynamics in an avian migrant

Ellen C. Martin, Thomas V. Riecke, Pierre-Alain Ravussin, Daniel Arrigo & Michael Schaub

Ecological Applications

Appendix S6

Figure S1. Graphical representation of our integrated population model for the European pied flycatcher populations in Baulmes and Corcelles-près-Concise, Switzerland. The model integrated nest box occupancy data (abundance model), reproductive data (fecundity model), and mark recapture data (survival model). Data is denoted by blue square boxes, while estimated parameters are denoted in peach ovals. In the fecundity model, $b_{a,s,t}$, $c_{a,s,t}$, and $f_{a,s,t}$ are the population-level total number of broods, clutch sizes, and number of fledglings for each stage class a in site s at time t (rec = one year old local recruits, im = immigrants, ad = adults two years and older). $\kappa_{a,s,t}$ and $\zeta_{a,s,t}$ are the average estimated clutch size and probability of fledging per individual egg, respectively. In the apparent survival model, $p_{j,a,s,t}$ reencounter probabilities per sex j, stage class a, site s, and time t. $m_{j,a,s,t}$ are the data of the m-array per sex j, stage class a, site s, and time t. $\phi_{j,a,s,t}$ is the apparent survival probability. In the abundance model, $y_{j,s,t}$ is the count of nest boxes occupied annually, with $\tau_{j,y}\tau y$ representing the residual error of the model. Values of $N_{j,a,s,t+1}$ are the estimated abundance per sex j, stage class a, site s, and year t, where rec = local recruits, ad = adults, im=immigrants.

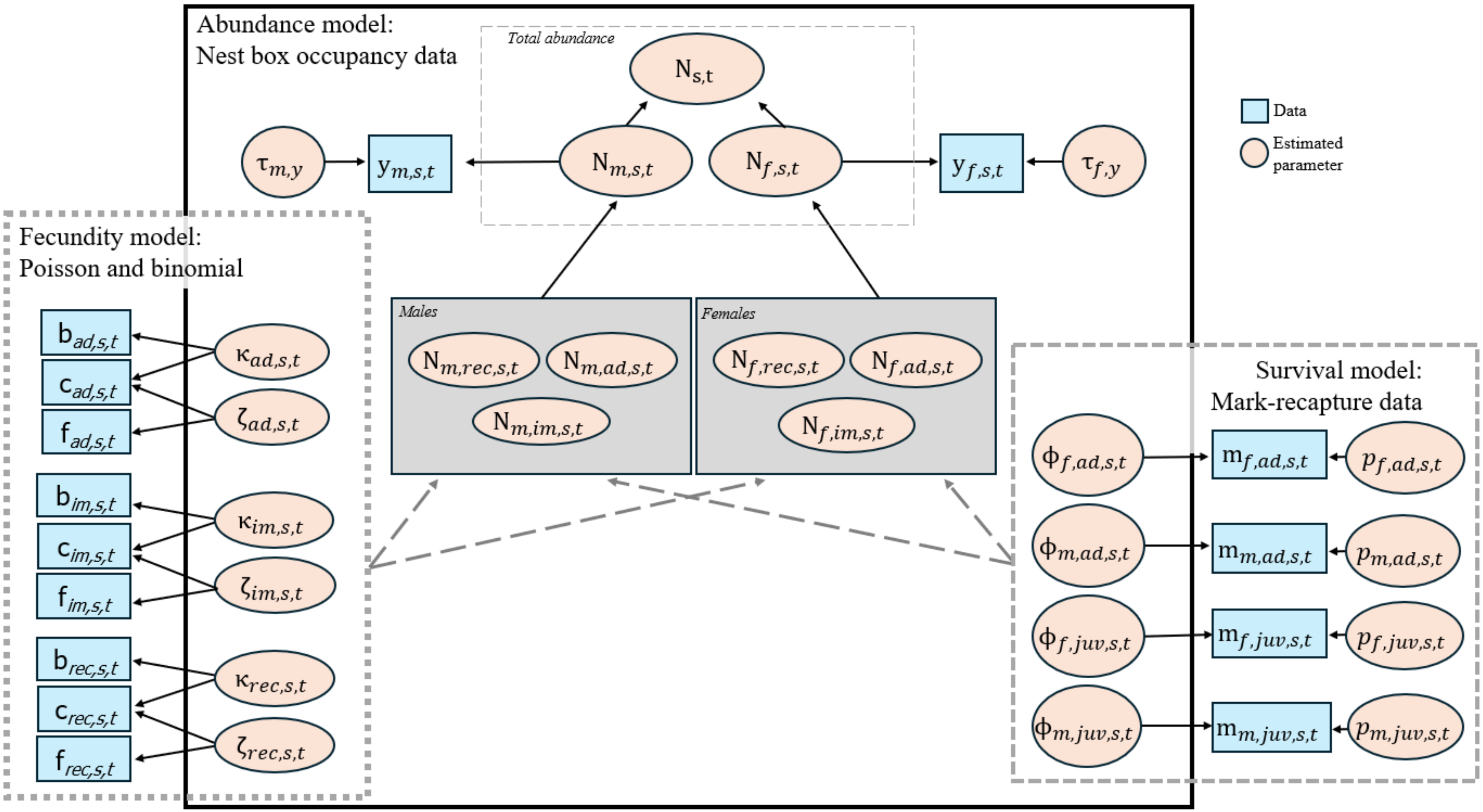
Abundance model:
Nest box occupancy data
Total abundance
N s,t
τ m,y
y m,s,t
N m,s,t
N f,s,t
y f,s,t
τ f,y
Data
Estimated parameter
Fecundity model:
Poisson and binomial
b ad,s,t
c ad,s,t
f ad,s,t
κ ad,s,t
ζ ad,s,t
b im,s,t
c im,s,t
f im,s,t
κ im,s,t
ζ im,s,t
b rec,s,t
c rec,s,t
f rec,s,t
κ rec,s,t
ζ rec,s,t
Males
N m,rec,s,t
N m,ad,s,t
N m,im,s,t
Females
N f,rec,s,t
N f,ad,s,t
N f,im,s,t
Survival model:
Mark-recapture data
ϕ f,ad,s,t
m f,ad,s,t
p f,ad,s,t
ϕ m,ad,s,t
m m,ad,s,t
p m,ad,s,t
ϕ f,juv,s,t
m f,juv,s,t
p f,juv,s,t
ϕ m,juv,s,t
m m,juv,s,t
p m,juv,s,t
