# Supplementary material for: Identifying the demographic pathways linking environmental covariates to population dynamics in an avian migrant
Source: Ecol Appl. 2026 Jan 5;36(1):e70166. doi: 10.1002/eap.70166 (PMC12770812; doi:10.1002/eap.70166)
Supplement: Supplementary file 7 — Appendix S7. [file EAP-36-e70166-s004.pdf]

Identifying the demographic pathways linking environmental covariates to population dynamics in an avian migrant

Ellen C. Martin, Thomas V. Riecke, Pierre-Alain Ravussin, Daniel Arrigo & Michael Schaub

Ecological Applications

Appendix S7

Table S1. Definitions of all parameters, data, and notation included in our IPM.

Parameter	Name	Definition	Equations
$N_{s,t}$	Total abundance	Total abundance of all ages and both sexes per site *s* and year *t*.	*NA*
$N_{j=f,s,t}$	Female abundance	Total abundance of females of all ages per site *s* and year *t*.	1
$N_{j=m,s,t}$	Male abundance	Total abundance of males of all ages per site *s* and year *t*.	5
$N_{j=f,rec,s,t}$	Female recruits	Total abundance of female recruits per site *s* and year *t*. Female recruits are birds born at year *t-1* in site *s* that survive until year *t* and return to site *s*.	2,3,6
$N_{j=f,ad,s,t}$	Female adults	Total abundance of female adults (≥2 years old) per site *s* and year *t*. Juvenile and immigrant females transition to female adults in year *t+1*.	2,3,6
$N_{j=f,im,s,t}$	Female immigrants	Total abundance of female immigrants per site *s* and year *t*. Female immigrants are birds born outside site *s* before *t* that have immigrated to site *s* in year *t*. They are assumed to be natal dispersers and one-year old.	2,3,4,6
$N_{j=m,rec,s,t}$	Male recruits	Total abundance of male recruits per site *s* and year *t*. Male recruits are birds born in year *t-1* in site *s* that survive until year *t* and return to site *s*.	6,7
$N_{j=m,ad,s,t}$	Male adults	Total abundance of male adults (≥2 years old) per site *s* and year *t*. Juvenile and immigrant males transition to male adults in year *t+1*.	6,7
$N_{j=m,im,s,t}$	Male immigrants	Total abundance of male immigrants per site *s* and year *t*. Male immigrants are birds born outside site *s* before *t* that have immigrated to site *s* in year *t*. They are assumed to be natal dispersers and one-year old.	7,8
$\kappa_{rec,s,t}$	Recruit clutch size	Average number of eggs laid per site *s* and year *t* for nests of female recruits.	2,6,14,15
$\kappa_{ad,s,t}$	Adult clutch size	Average number of eggs laid per site *s* and year *t* for nests of female adults.	2,6,14,15

$\kappa_{im,s,t}$	Immigrant clutch size	Average number of eggs laid per site *s* and year *t* for nests of female immigrants.	2,6,14,15
$\zeta_{rec,s,t}$	Recruit probability of fledging	Probability that an egg survives to fledge from nests of female recruits in site *s* in year *t*.	2,6,16
$\zeta_{ad,s,t}$	Adult probability of fledging	Probability that an egg survives to fledge from nests of female adults in site *s* in year *t*.	2,6,16
$\zeta_{im,s,t}$	Immigrant probability of fledging	Probability that an egg survives to fledge from nests of female immigrants in site *s* in year *t*.	2,6,16
$\phi_{j=f,juv,s,t}$	Apparent survival female juvenile	Probability that juvenile females in site *s* survive from post-fledging period in year *t* until the prebreeding census in year *t+1* and do not permanently emigrate from site *s*.	2
$\phi_{j=f,ad,s,t}$	Apparent survival female adult	Probability that adult females in site *s* survive from year *t* until the prebreeding census in year *t+1* and do not permanently emigrate from site *s*.	3
$\phi_{\mathrm{j}=m,juv,s,t}$	Apparent survival male juvenile	Probability that juvenile males in in site *s* survive from post-fledging period in year *t* until the prebreeding census in year *t+1* and do not permanently emigrate from site *s*.	6
$\phi_{j=m,ad,s,t}$	Apparent survival male adult	Probability that adult males in site *s* survive from year *t* until the prebreeding census in year *t+1* and do not permanently emigrate from site *s*.	7
$\omega_{\mathrm{j},s,}$	Number of immigrants	Expected number of female (*j=f*) and male (*j=m*) immigrants in a site *s* in year *t*.	4,8,17
$\pi_{j,a,s}$	Reencounter vectors	Vector for females (*j=f*) and males (*j=m*) containing probabilities expressing when individuals released of sex *j*, stage *a*, site *s* in year *t* were reencountered	Appendix S9

		for the first time as a function of apparent survival and reencounter probabilities.	
$p_{j,a,s,t}$	Reencounter probability	The probability that a banded individual of sex *j*, in stage *a,* at site *s,* in year *t* is recaptured and identified, given that it is present at site *s* in year *t.*	9
$\mu_{p,j,a,s}$	Reencounter probability mean	Reencounter probability: sex-, age- and site-specific mean.	9
$\varepsilon_{p,j,a,s,t}$	Reencounter probability temporal variation	Annual deviation from the mean reencounter probability for sex *j*, stage *a*, site *s* and year *t.*	9,10
$\mu_{\phi,j,a,s}$	Mean survival	Mean annual apparent survival for individuals of sex *j*, stage *a* in site *s* across all years.	11
$\varepsilon_{\phi,j,a,s,t}$	Survival temporal variation	Annual deviation from the mean survival probability for sex *j*, stage *a*, site *s* and year *t.*	11,12
$\beta_{\phi,\text{cov},a}$	Covariate effect	Effect of the covariate for stage class *a* on survival on the logistic scale.	13
$\beta_{\zeta,\text{cov},a}$	Covariate effect	Effect of the covariate for stage class *a* on probability of fledging on the logistic scale.	Appendix S10
$\mu_{\kappa,a,s}$	Clutch size mean	Age- *a* and site-specific *s* clutch size mean across all years.	15
$\varepsilon_{\kappa,a,s,t}$	Clutch size temporal variation	Annual deviation from the mean clutch size per stage class *a*, site *s* and year *t*.	15
$\mu_{\zeta,a,s}$	Mean probability of fledging	Stage and site-specific mean probability of fledging for individual eggs laid by females of stage *a* in site *s.*	Appendix S9

$\tau_{y,s}$	Residual error	Residual error of the integrated population model containing observation errors of the population counts and potential lack of fit of the process model for sites *s*.	18
$NID_{s,t}$	Nest initiation date	The average date (Julian) that the first egg was laid in all nests in site *s* in year *t*.	Appendix S10
MastYear_t	Mast year	Binary variable representing if a summer period was classified as a mast year (1) or no-mast year (0). We used full-and-partial mast years of Oak trees (*Quercus spp*.) near the study populations as a proxy for dormouse abundance in year *t* and in year *t+1* (i.e., a one-year time lag). Data on masting events was available for the years 1988 until 2020 from the WSL MastWeb application (WSL MastWeb Application. Swiss Federal Institute for Forest, Snow and Landscape Research WSL. Accessed 18 July 2024). We classified years as "no mast" or "mast" years based on the classification of the closest *Quercus spp*. to the study site each year. Partial and half-mast years were grouped together into the mast year category. MastYear_{t-1} = time-lagged effect of mast-year.	Appendix S10
Summer.Temp_t	Mean summer temperature	The average temperature from daily 24-hour periods for the entire duration of the designated "summer" period (nest initiation/incubation, hatchling, and post-fledging periods combined).	Appendix S10
NII.Mean.Temp_t<br>H.Mean.Temp_t<br>PF.Mean.Temp_t	Mean period temperature	The average temperature from daily 24-hour periods for each of the breeding periods in year *t.* Abbreviations: NII=Nest Initiation/Incubation, H=Hatchling, and PF=Post-fledging periods.	Appendix S10
NII.Min.Temp_t<br>H.Min.Temp_t	Minumum period temperature	The average minimum temperature from daily 24-hour periods for each of the breeding periods in year *t.*	Appendix S10

$\mathrm{PF.Min.Temp}_t$			
$\mathrm{NII.Max.Temp}_t$<br>$\mathrm{H.Max.Temp}_t$<br>$\mathrm{PF.Max.Temp}_t$	Maximum period temperature	The average maximum temperature from daily 24-hour periods for each of the breeding periods in year *t*.	Appendix S10
$\mathrm{Summer.Precip}_t$	Mean summer precipitation	The cumulative precipitation from daily 24-hour periods for the entire duration of the designated "summer" period (All dates inclusive of nest initiation/incubation, hatchling, and post-fledging periods).	Appendix S10
$\mathrm{NII.Precip}_t$<br>$\mathrm{H.Precip}_t$<br>$\mathrm{PF.Precip}_t$	Mean period precipitation	The cumulative precipitation from daily 24-hour periods for each of the breeding periods in year *t*.	Appendix S10
NDVI	Mean NDVI	The mean NDVI value for the whole overwintering period which included November, December of year *t* and January, February of year *t+1*.	Appendix S10
**Indexing:**			
a	Stage class	For fecundity, 3 stage classes (recruits [one-year old local recruits], immigrants [one-year old immigrants], adults [two-years old and older]). For survival, 2 stage classes (juvenile [one-year olds], adult [two-years and older]).	
s	Site	Sites (Baulmes or Corcelles), used interchangeably with *"populations"*.	
j	Sex	Sex, females (*j=f*) or males (*j=m*).	
t	Year	Year; *1, 2, …. T*.	

f	Female	Female	
m	Male	Male	
B	Baulmes	Site Baulmes	
C	Corcelles	Site Corcelles-près-Concise	
**Data:**			
$\mathrm{m}_{j,a,s,t}$	m-array	Data of the m-array for sex *j* of stage *a* at site *s* in year *t*.	Appendix S10
$\mathrm{R}_{j,a,s,t}$	Released individuals	The total number of individuals of sex *j*, stage *a* released at site *s* for each year *t* of nest box monitoring.	Appendix S10
$\mathrm{Covariate}_{s,t}$	Covariate value	Observed covariate value at site *s* in year *t*. When subscript *s* is absent, the covariate was the same value across sites.	13
$\mathrm{b}_{a,s,t}$	Number of broods	Sum of the number of broods (i.e., nesting attempts) across all nest boxes per female stage class *a*, site *s*, and year *t*. Population-level data.	14
$\mathrm{c}_{a,s,t}$	Clutch size	Sum of all eggs across all nests per female stage class *a*, site *s*, and year *t*. Population-level data.	14,16
$\mathrm{f}_{a,s,t}$	Number of fledglings	Sum of the number of fledglings from all broods at the population level per female stage class *a*, site *s*, and year *t*. Population-level data.	16
$\mathrm{y}_{j,s,t}$	Count of individuals	Number of individuals of sex *j* in site *s*, calculated from the number of occupied nest boxes at each site in each year *t* as proxy.	18
