# Supplementary material for: Identifying the demographic pathways linking environmental covariates to population dynamics in an avian migrant
Source: Ecol Appl. 2026 Jan 5;36(1):e70166. doi: 10.1002/eap.70166 (PMC12770812; doi:10.1002/eap.70166)

Identifying the demographic pathways linking environmental covariates to population dynamics in an avian migrant

Ellen C. Martin, Thomas V. Riecke, Pierre-Alain Ravussin, Daniel Arrigo & Michael Schaub

Ecological Applications

Appendix S8

Figure S1. Temporal correlation of the probability of fledging (ρ ζ) between the three stage classes' (Im = immigrants, Ad = adults, Rec = recruit) for Baulmes (top) and Corcelles (bottom).

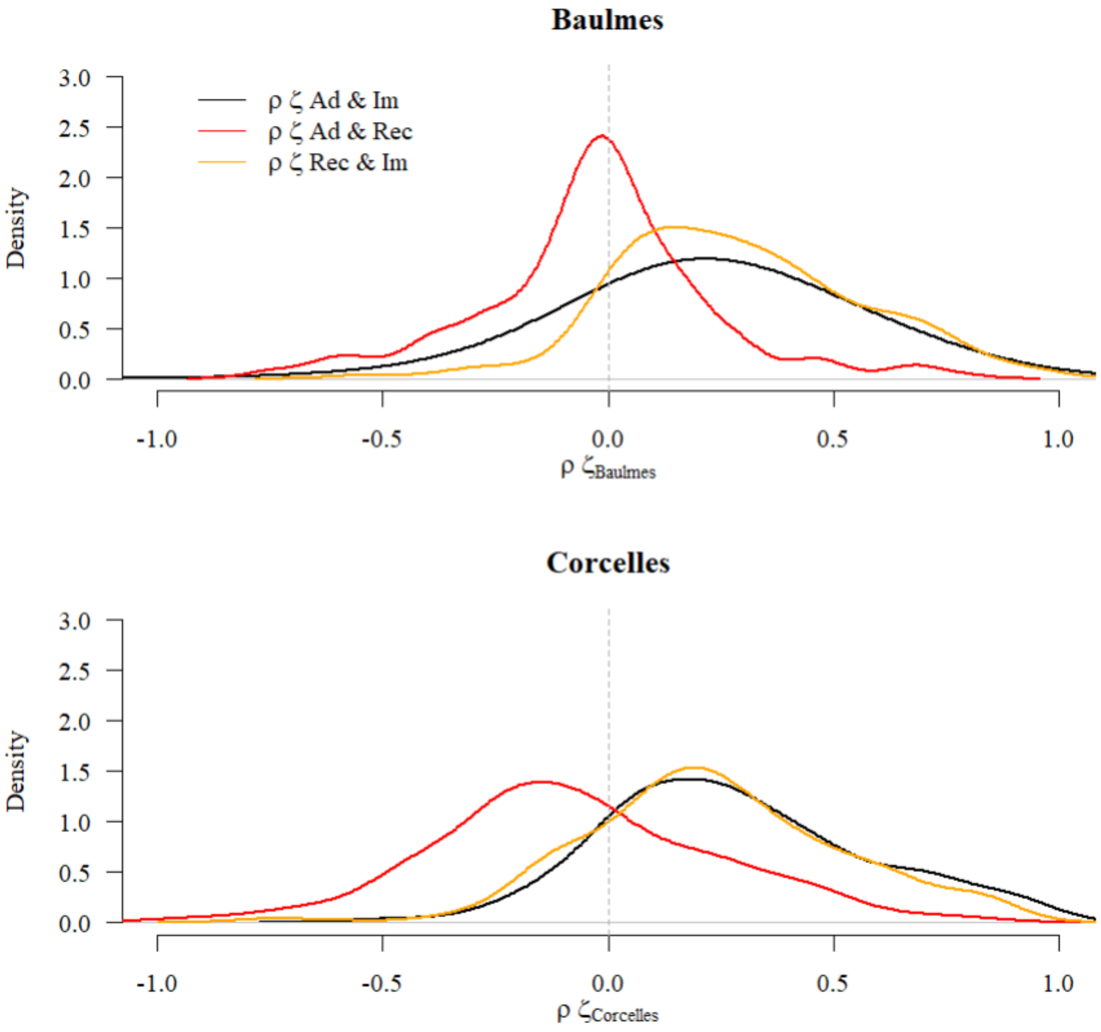

Supplement: Supplementary file 8 — Appendix S8. [file EAP-36-e70166-s003.pdf]
