# Supplementary material for: Identifying the demographic pathways linking environmental covariates to population dynamics in an avian migrant
Source: Ecol Appl. 2026 Jan 5;36(1):e70166. doi: 10.1002/eap.70166 (PMC12770812; doi:10.1002/eap.70166)
Supplement: Supplementary file 9 — Appendix S9. [file EAP-36-e70166-s008.pdf]

Identifying the demographic pathways linking environmental covariates to population dynamics in an avian migrant

Ellen C. Martin, Thomas V. Riecke, Pierre-Alain Ravussin, Daniel Arrigo & Michael Schaub

Ecological Applications

Appendix S9

Appendix S9. Further model details.

Section S1: Model implementation specifications and prior information.

I. From Methods: Data Analysis: Estimation of apparent survival probabilities

a. $p_{j,a,s,t}$

Recapture probability (Equation 9):

Linear model:

$$logit(p_{j,a,s,t}) = \mu_{p,j,a,s} + \varepsilon_{p,j,a,s,t}$$

Priors:

$$\mu_{p,j,a,s} \sim logistic(0, 1)$$
$$\varepsilon_{p,j,a,s,t} \sim normal(0,\ \tau_{p,j,a,s})$$
$$\tau_{p,j,a,s} = \frac{1}{\sigma^2_{p,j,a,s}}$$
$$\sigma_{p,j,a,s} \sim uniform(0, 6)$$

b. $\phi_{j,a,s,t}$

Apparent survival probability (Equation 11):

The apparent survival parameter ($\phi_{j,a,s,t}$) described the probability that an individual of sex j in stage class a survived from one breeding season to the next and returned to site s. We separated capture histories of individuals into two sexes (females and males), and further separated them into two stage classes (juveniles and adults). We formatted the capture histories for individuals in these four classes into unique m-arrays[1,2]. The m-array table format provided a summary across populations for each of the stage and sex classes of how many individuals were released each year and the year they were reencountered post-release.

Linear model:

$$logit(\phi_{j,a,s,t}) = \mu_{\phi,j,a,s} + \varepsilon_{\phi,j,a,s,t}$$

Priors:

$$\mu_{\phi,j,a,s} \sim logistic(0, 1)$$
$$\varepsilon_{\phi,j,a,s,t} \sim normal(0,\ \tau_{\phi,j,s})$$
$$\tau_{\phi,j,s} = \frac{1}{\sigma^2_{\phi,j,s}}$$
$$\sigma_{\phi,j,s} \sim uniform(0, 1)$$

There are sex-specific linear models and priors for males that are formulated in the same way as specified above.

The likelihoods for the capture-recapture data in the m-array for sex j,

[1] Schaub, M., & Kéry, M. (2022). Integrated population models: Theory and ecological applications with R and JAGS. Chapter 4. Academic Press.·

[2] Williams, B. K., Nichols, J. D., & Conroy, M. J. (2002). Analysis and management of animal populations. Academic press.

$$m_{j,a,s,t} \sim multinomial(\pi_{j,a,s}, R_{j,a,s,t}).$$

$m_{j,a,s,t}$ was the sex-, stage- and site-specific data of the m-array, where each row of the m-array represented a release year *t*. The parameters $\pi_{j,a,s}$ contained probabilities expressing when individuals that were released in year *t* were recaptured as a function of apparent survival probability $(\phi_{j,a,s,t})$ and recapture probability $(p_{j,a,s,t})$. $R_{j,a,s,t}$ was the total numbers of individuals released for each sex, stage, and site in year *t*.

c. $\beta_{\phi,cov,a}$
Slope of the effect of the covariate *cov* for stage class *a* (Equation 13):
Linear model:

$$logit(\phi_{f,a,s,t}) = \mu_{\phi f,a,s} + \beta_{\phi,cov,a}\text{Covariate}_t + \varepsilon_{\phi,f.a,s,t}$$

Priors:

$$\beta_{\phi,cov,a} \sim normal(0, 0.1)$$

$$\mu_{\phi,f,a,s} \sim logistic(0, 1)$$
$$\varepsilon_{\phi,f,a,s,t} \sim normal(0, \tau_{\phi,f,s})$$
$$\tau_{\phi,f,s} = \frac{1}{\sigma^2_{\phi,j,s}}$$
$$\sigma_{\phi,j,s} \sim uniform(0, 1)$$

II. From Methods: Data Analysis: Estimation of fecundity

a. $\kappa_{a,s,t}$
Clutch size (Equations 14 and 15):
Linear model:

$$c_{a,s,t} \sim Poisson(\kappa_{a,s,t} \times b_{a,s,t}).$$
$$log(\kappa_{a,s,t}) = \mu_{\kappa,a,s} + \varepsilon_{\kappa,a,s,t}$$

Priors:

$$\mu_{\kappa,a,s} \sim normal(0, 0.001),$$
$$\varepsilon_{\kappa,a,s,t} \sim normal(0, \tau_{\kappa,\varepsilon,a,s})$$
$$\tau_{\kappa,\varepsilon,a,s} = \frac{1}{\sigma^2_{\kappa,\varepsilon,a,s}}$$
$$\sigma_{\kappa,\varepsilon,a,s} \sim uniform(0,1)$$

b. $\zeta_{a,s,t}$
Probability to fledge (Equation 16):

We accounted for correlated probability to fledge following the approach of Chen & Dunson (2003)[3] and Fay et al. (2021[4], 2022[5]) wherein we used correlated random effects and modeled the variance-covariance matrix using Cholesky decomposition with parameter expansion. See Appendix S3 of Fay et al. 2021[4] for tutorial on Cholesky decomposition and parameter expansion.

Linear model:

$$f_{a,s,t} \sim Binomial(c_{a,s,t}, \zeta_{a,s,t}).$$
$$logit(\zeta_{a,s,t}) = \mu_{\zeta,a,s,t} + \varepsilon_{\zeta,a,s,t}$$

where $\mu_{\zeta,a,s,t}$ is the mean stage-specific estimate of probability of fledging and $\varepsilon_{\zeta,a,s,t}$ is the correlated random effect term.

Priors:

$$\mu^{\zeta}_{a,s} \sim Logistic(0,1)$$

We modeled yearly variation in fledging success parameters separately for each site using a multivariate normal distribution with uniform priors on standard deviations and pairwise correlations. We estimated pairwise correlations (ρ_{12}, ρ_{13}, ρ_{23}) among the three demographic parameters using a reparameterized covariance matrix, where:

$$\sigma_{\rho} \sim uniform(0, 5)$$
$$\rho \sim uniform(-1, 1)$$

Like with survival, the covariates we were interested in testing had varying degrees of correlation, with some highly correlated (Appendix S5). We therefore ran separate IPMs for each covariate of interest to determine which covariate had an important influence on probability of fledging.

III. From Methods: Data Analysis: Estimation of immigrants

a. $\omega_{j,s}$

Estimation of immigrants (Equation 17):

$$N_{j,im,s,t} \sim Poisson(\omega_{j,s}),$$

Prior:

$$\omega_{j,s} \sim \text{Log Normal}(0, 1)$$

---

[3] Chen, Z., & Dunson, D. B. (2003). Random effects selection in linear mixed models. Biometrics, 59(4), 762–769.

[4] Fay, R., Authier, M., Hamel, S., Jenouvrier, S., van de Pol, M., Cam, E., Gaillard, J.-M., Yoccoz, N. G., Acker, P., Allen, A., Aubry, L. M., Bonenfant, C., Caswell, H., Coste, C. F. D., Larue, B., Le Coeur, C., Gamelon, M., Macdonald, K. R., Moiron, M., … Sæther, B.-E. (2022). Quantifying fixed individual heterogeneity in demographic parameters: Performance of correlated random effects for Bernoulli variables. *Methods in Ecology and Evolution*, 13, 91–104. https://doi.org/10.1111/2041-210X.13728

[5] Fay, R., Hamel, S., van de Pol, M., Gaillard, J.-M., Yoccoz, N.G., Acker, P., et al. (2022) Temporal correlations among demographic parameters are ubiquitous but highly variable across species. *Ecology Letters*, 25, 1640–1654. Available from: https://doi.org/10.1111/ele.14026

IV. From Methods: Data Analysis: Estimation of population sizes

a. $y_{j,s,t}$

Estimation of population sizes (Equation 18):

Model:

$$y_{j,s,t} \sim \log Normal\left(N_{j,s,t}, \tau_{y,s}\right)$$

Prior:

$$\tau_{y,s} = \frac{1}{\sigma_{y,s}^2}$$

$$\sigma_{y,s} \sim \text{Uniform}(0.1, 100)$$

Section S2: Decomposition of variance.

Following Knape et al. 2023[6], we decomposed the variation in realized population growth rates into contributions from environmental factors for females. In brief, this method involved splitting the linear predictor into different components (i.e., linear predictor components: "LPC") that each represented the site and year varying environmental or phenological covariates that had an 85% Credible Interval that did not overlap 0.

LPC contribution from environmental covariates:

$$LPC_{a,s,t}^{\text{ENV},\phi,\text{f}} = \boldsymbol{E}_{a,s}^{\phi,f} \times \beta_{\phi,cov,a},$$

where $\boldsymbol{E}_{a,s}^{\phi,f}$ was a matrix of dimensions: environmental covariates by number of years of each covariate that was significantly different from zero for each demographic rate tested in part 1.

$$\boldsymbol{E}_{a,s}^{\phi,f} = \begin{bmatrix} cov_{1,t} & cov_{2,t} \\ cov_{1,t+1} & cov_{2,t+2} \\ cov_{1,t+2} & cov_{2,t+3} \\ \cdots & \cdots \end{bmatrix}, \text{ and}$$

$$\beta_{\phi,cov,a} \sim Normal\ (0,1).$$

LPC contribution from error:

$$LPC_{a,s,t}^{\text{ERROR},\phi,\text{f}} = \mu_{\phi,f,a,s} + \varepsilon_{\phi,f,a,s,t}$$

LPC total contribution:

$$logit\left(\phi_{f,a,s,t}\right) = \sum LPC_{a,s,t}^{\text{ENV},\phi,\text{f}} + \sum LPC_{a,s,t}^{\text{ERROR},\phi,\text{f}}$$
