# Supplementary material for: Identifying the demographic pathways linking environmental covariates to population dynamics in an avian migrant
Source: Ecol Appl. 2026 Jan 5;36(1):e70166. doi: 10.1002/eap.70166 (PMC12770812; doi:10.1002/eap.70166)
Supplement: Supplementary file 10 — Appendix S10. [file EAP-36-e70166-s007.pdf]

Identifying the demographic pathways linking environmental covariates to population dynamics in an avian migrant

Ellen C. Martin, Thomas V. Riecke, Pierre-Alain Ravussin, Daniel Arrigo & Michael Schaub

Ecological Applications

Appendix S10

Table S1. List of IPMs fitted. Some environmental variables were highly correlated and therefore covariates run in separate models (Appendix 2). Model names and numbers are presented with the corresponding formulas for apparent survival ($\phi_{f,a,s,t}$) and probability of fledging ($\zeta_{a,s,t}$). Bolded parameters indicate the parameters added to base model and correspond to the covariate(s) being assessed. See Appendix S3 for parameter definitions.

Model Number & Name	Model
1. Base model, excluding covariates	$logit(\phi_{f,a,s,t}) = \mu_{\phi,f,a,s} + \varepsilon_{\phi,f,a,s,t}$<br>$logit(\zeta_{a,s,t}) = \mu_{\zeta,a,s} + \varepsilon_{\zeta,a,s,t}$
2. Mean Temp, Full summer	$logit(\phi_{f,a,s,t}) = \mu_{\phi,f,a,s} + \boldsymbol{\beta_{\phi,summer.temp,a}} \times \mathbf{Summer.Temp_t} + \varepsilon_{\phi,f,a,s,t}$<br>$logit(\zeta_{a,s,t}) = \mu_{\zeta,a,s} + \boldsymbol{\beta_{\zeta,summer.temp,a}} \times \mathbf{Summer.Temp_t} + \varepsilon_{\zeta,a,s,t}$
3. Mean Temp, Periods	$logit(\phi_{f,a,s,t}) = \mu_{\phi,f,a,s} + \boldsymbol{\beta_{\phi,NII.mean.temp,a}} \times \mathbf{NII.Mean.Temp_t} + \boldsymbol{\beta_{\phi,H.mean.temp,a}} \times \mathbf{H.Mean.Temp_t} + \boldsymbol{\beta_{\phi,PF.mean.temp,a}} \times \mathbf{PF.Mean.Temp_t} + \varepsilon_{\phi,f,a,s,t}$<br>$logit(\zeta_{a,s,t}) = \mu_{\zeta,a,s} + \boldsymbol{\beta_{\zeta,NII.mean.temp,a}} \times \mathbf{NII.Mean.Temp_t} + \boldsymbol{\beta_{\zeta,H.mean.temp,a}} \times \mathbf{H.Mean.Temp_t} + \boldsymbol{\beta_{\zeta,PF.mean.temp,a}} \times \mathbf{PF.Mean.Temp_t} + \varepsilon_{\zeta,a,s,t}$
4. Min Temp, Periods	$logit(\phi_{f,a,s,t}) = \mu_{\phi,f,a,s} + \boldsymbol{\beta_{\phi,NII.min.temp,a}} \times \mathbf{NII.MinTemp_t} + \boldsymbol{\beta_{\phi,H.min.temp,a}} \times \mathbf{H.MinTemp_t} + \boldsymbol{\beta_{\phi,PF.min.temp,a}} \times \mathbf{PF.MinTemp_t} + \varepsilon_{\phi,f,a,s,t}$<br>$logit(\zeta_{a,s,t}) = \mu_{\zeta,a,s} + \boldsymbol{\beta_{\zeta,NII.min.temp,a}} \times \mathbf{NII.MinTemp_t} + \boldsymbol{\beta_{\zeta,H.min.temp,a}} \times \mathbf{H.MinTemp_t} + \boldsymbol{\beta_{\zeta,PF.min.temp,a}} \times \mathbf{PF.MinTemp_t} + \varepsilon_{\zeta,a,s,t}$
5. Max Temp, Periods	$logit(\phi_{f,a,s,t}) = \mu_{\phi,f,a,s} + \boldsymbol{\beta_{\phi,NII.max.temp,a}} \times \mathbf{NII.MaxTemp_t} + \boldsymbol{\beta_{\phi,H.max.temp,a}} \times \mathbf{H.MaxTemp_t} + \boldsymbol{\beta_{\phi,PF.max.temp,a}} \times \mathbf{PF.MaxTemp_t} + \varepsilon_{\phi,f,a,s,t}$<br>$logit(\zeta_{a,s,t}) = \mu_{\zeta,a,s} + \boldsymbol{\beta_{\zeta,NII.max.temp,a}} \times \mathbf{NII.MaxTemp_t} + \boldsymbol{\beta_{\zeta,H.max.temp,a}} \times \mathbf{H.MaxTemp_t} + \boldsymbol{\beta_{\zeta,PF.max.temp,a}} \times \mathbf{PF.MaxTemp_t} + \varepsilon_{\zeta,a,s,t}$
6. Cum. Precip, Full summer	$logit(\phi_{f,a,s,t}) = \mu_{\phi,f,a,s} + \boldsymbol{\beta_{\phi,summer.precip,a}} \times \mathbf{Summer.Precip_t} + \varepsilon_{\phi,f,a,s,t}$<br>$logit(\zeta_{a,s,t}) = \mu_{\zeta,a,s} + \boldsymbol{\beta_{\zeta,summer.precip,a}} \times \mathbf{Summer.Precip_t} + \varepsilon_{\zeta,a,s,t}$
7. Cum. Precip, Periods	$logit(\phi_{f,a,s,t}) = \mu_{\phi,f,a,s} + \boldsymbol{\beta_{\phi,NII.precip,a}} \times \mathbf{NII.Precip_t} + \boldsymbol{\beta_{\phi,H.precip,a}} \times \mathbf{H.Precip_t} + \boldsymbol{\beta_{\phi,PF.precip,a}} \times \mathbf{PF.Precip_t} + \varepsilon_{\phi,f,a,s,t}$<br>$logit(\zeta_{a,s,t}) = \mu_{\zeta,a,s} + \boldsymbol{\beta_{\zeta,NII.precip,a}} \times \mathbf{NII.Precip_t} + \boldsymbol{\beta_{\zeta,H.precip,a}} \times \mathbf{H.Precip_t} + \boldsymbol{\beta_{\zeta,PF.precip,a}} \times \mathbf{PF.Precip_t} + \varepsilon_{\zeta,a,s,t}$

8. Nest initiation date (*NID*)	$logit(\phi_{f,a,s,t}) = \mu_{\phi,f,a,s} + \varepsilon_{\phi,f,a,s,t}$<br>$logit(\zeta_{a,s,t}) = \mu_{\zeta,a,s} + \boldsymbol{\beta_{\zeta,NID,a}} \times \mathbf{NestInitDate_{a,s,t}} + \varepsilon_{\zeta,a,s,t}$
9. Mast Year(*t*)	$logit(\phi_{f,a,s,t}) = \mu_{\phi,f,a,s} + \boldsymbol{\beta_{\phi,Mast.year,a}} \times \mathbf{MastYear_{t}} + \varepsilon_{\phi,f,a,s,t}$<br>$logit(\zeta_{a,s,t}) = \mu_{\zeta,a,s} + \boldsymbol{\beta_{\zeta,Mast.year,a}} \times \mathbf{MastYear_{t}} + \varepsilon_{\zeta,a,s,t}$
10. Mast Year time lag (*t-1*)	$logit(\phi_{f,a,s,t}) = \mu_{\phi,f,a,s} + \boldsymbol{\beta_{\phi,Mast.year.t-1,a}} \times \mathbf{MastYear_{t-1}} + \varepsilon_{\phi,f,a,s,t}$<br>$logit(\zeta_{a,s,t}) = \zeta_{a,s,t} + \boldsymbol{\beta_{\zeta,Mast.year.t-1,a}} \times \mathbf{MastYear_{t-1}} + \varepsilon_{\zeta,a,s,t}$
11. NDVI	$logit(\phi_{f,a,s,t}) = \mu_{\phi,f,a,s} + \boldsymbol{\beta_{\phi,NDVI,a}} \times \mathbf{NDVI_{t-1}} + \varepsilon_{\phi,f,a,s,t}$<br>$logit(\zeta_{a,s,t}) = \mu_{\zeta,a,s} + \boldsymbol{\beta_{\zeta,NDVI,a}} \times \mathbf{NDVI_{t-1}} + \varepsilon_{\zeta,a,s,t}$
12. Final global model	$logit(\phi_{f,a,s,t}) = \mu_{\phi,f,a,s} + \beta_{\phi,NII.precip,juv} \times NII.Precip_t + \beta_{\phi,H.precip,juv} \times H.Precip_t + \beta_{\phi,NII.precip,ad} \times NII.Precip_t + \beta_{\phi,H.precip,ad} \times H.Precip_t + \beta_{\phi,\mathrm{P.F.temp,juv}} \times PF.Temp_t + \varepsilon_{\phi,f,a,s,t}$<br>$logit(\zeta_{a,s,t}) = \mu_{\zeta,a,s} + \beta_{\zeta,\mathrm{NID},rec} \times NID_{rec,s,t} + \beta_{\zeta,\mathrm{NDVI},im,t} \times NDVI_{t-1} + \beta_{\zeta,\mathrm{MastYearTimeLag},rec,t} \times MastYear_{t-1} + \beta_{\zeta,\mathrm{H}.min.temp,imm,t} \times H.MinTemp_t + \beta_{\zeta,\mathrm{summer.temp},ad,t} \times SummerTemp_t + \varepsilon_{\zeta,a,s,t}$.
