# Supplementary material for: Identifying the demographic pathways linking environmental covariates to population dynamics in an avian migrant
Source: Ecol Appl. 2026 Jan 5;36(1):e70166. doi: 10.1002/eap.70166 (PMC12770812; doi:10.1002/eap.70166)
Supplement: Supplementary file 11 — Appendix S11. [file EAP-36-e70166-s001.pdf]

Identifying the demographic pathways linking environmental covariates to population dynamics in an avian migrant

Ellen C. Martin, Thomas V. Riecke, Pierre-Alain Ravussin, Daniel Arrigo & Michael Schaub

Ecological Applications

Appendix S11

Figure S1. We used the Brooks and Gelman diagnostic[1] to assess the convergence of the MCMC simulations on the base IPM (i.e., not including covariates; Model #1, Appendix 10). These were all below 1.3. (A). Model fit was assessed by posterior predictive p-values[3] (B-F). The fit was deemed acceptable for the aspects of the model that we investigated.

We assessed MCMC convergence using the Brooks and Gelman R-hat diagnostic (Brooks & Gelman, 1998), which compares within- and between-chain variance to determine whether the chains have converged to the same posterior distribution. R-hat values close to 1 indicate convergence. All values were below 1.3, with the vast majority below 1.1. Values above 1.3 can suggest poor mixing or insufficient chain length, which, if ignored, may lead to biased or unreliable posterior estimates.

To assess model fit, we conducted posterior predictive checks using test statistics on observed versus replicate datasets simulated from the posterior predictive distribution (Gelman et al., 1996)[2]. Posterior predictive p-values close to 0.5 suggest that the model is capturing the data-generating process well, while values near 0 or 1 indicate poor fit. For our model, posterior predictive p-values ranged from 0.11 to 0.47 across model components, indicating that the model reproduces key features of the data adequately. The lack of extreme posterior predictive p-values supports the conclusion that the base IPM (Model #1) provides a reasonable fit to the observed data. We also note that posterior predictive checks are particularly valuable in hierarchical models like IPMs, where traditional goodness-of-fit metrics are often unavailable or inappropriate.

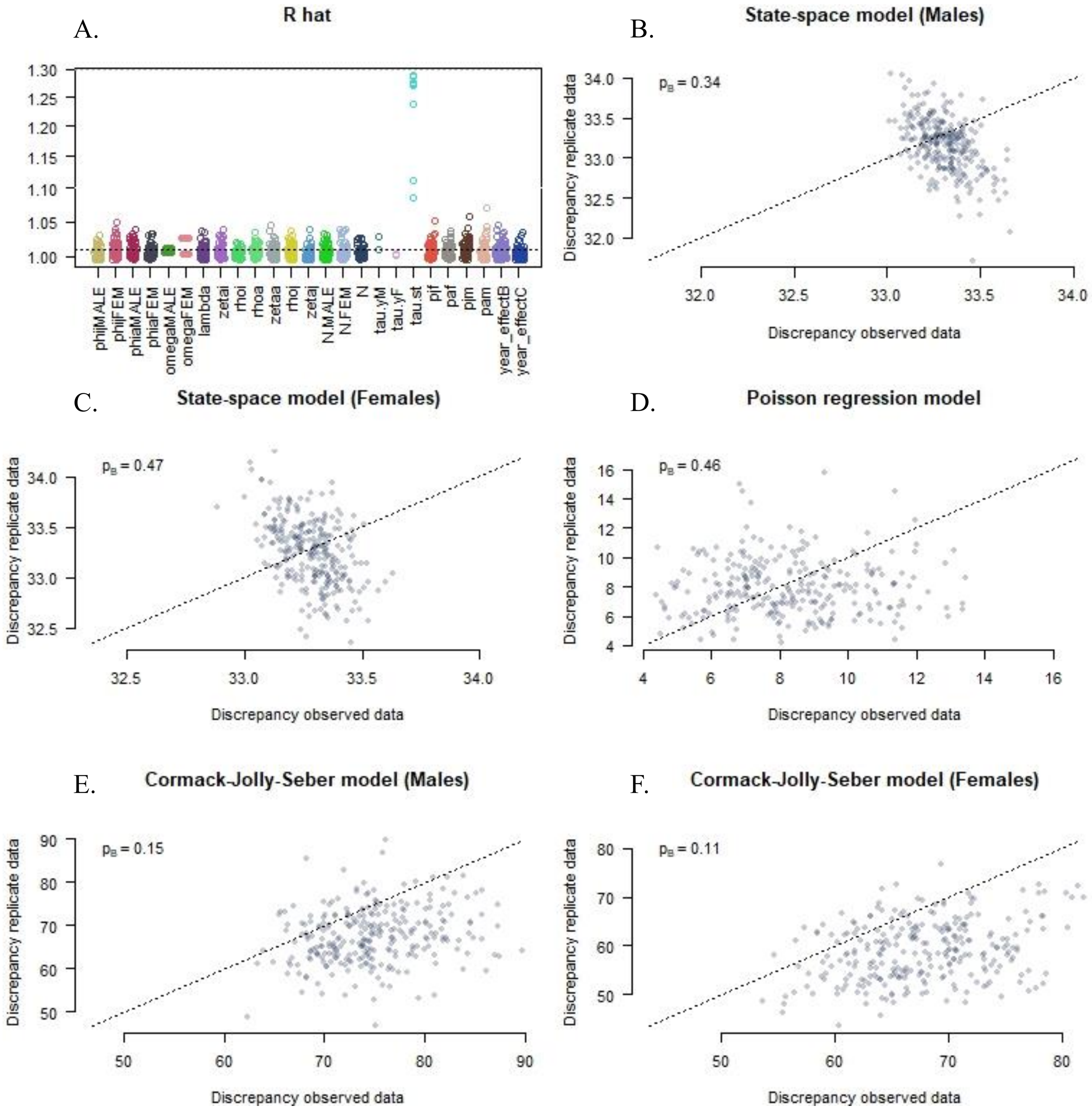

1. Brooks, S. P., & Gelman, A. (1998). General methods for monitoring convergence of iterative simulations. Journal of Computational and Graphical Statistics, 7(4), 434–455.
2. Gelman, A., Meng, X. L., & Stern, H. (1996). Posterior predictive assessment of model fitness via realized discrepancies. Statistica sinica, 733-760.
3. Schaub, M., & Kéry, M. (2022). Integrated population models: Theory and ecological applications with R and JAGS. Academic Press.
