# Supplementary material for: Identifying the demographic pathways linking environmental covariates to population dynamics in an avian migrant
Source: Ecol Appl. 2026 Jan 5;36(1):e70166. doi: 10.1002/eap.70166 (PMC12770812; doi:10.1002/eap.70166)

Identifying the demographic pathways linking environmental covariates to population dynamics in an avian migrant

Ellen C. Martin, Thomas V. Riecke, Pierre-Alain Ravussin, Daniel Arrigo & Michael Schaub

Ecological Applications

Appendix S12

Figure S1. Estimates of the number of female (A) and male (B) immigrants in Baulmes (in blue) and Corcelles (in orange) from the mean of the posterior distribution from the base model excluding environmental covariates.

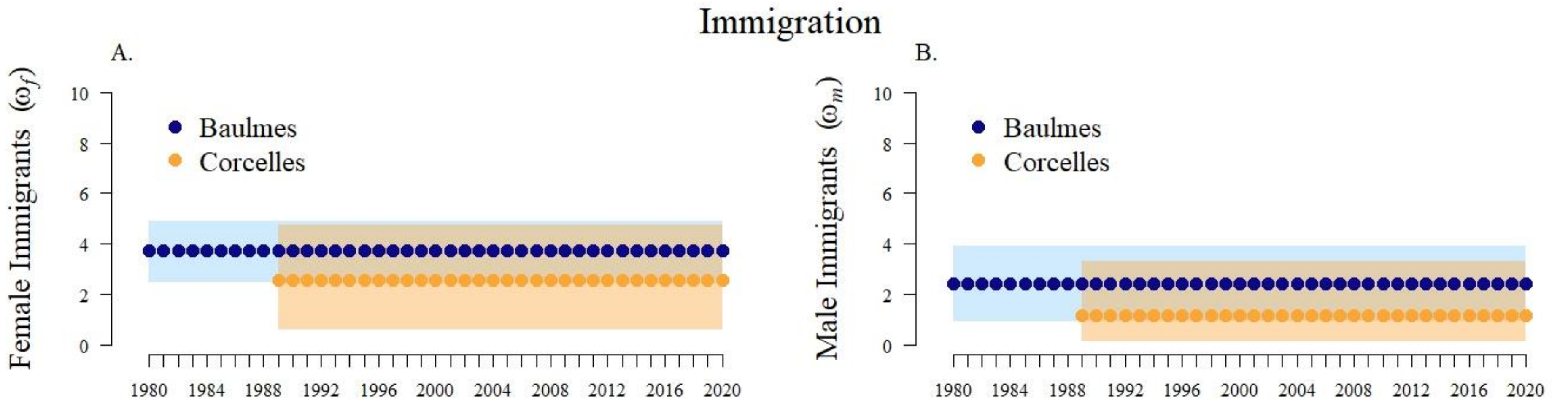

Supplement: Supplementary file 12 — Appendix S12. [file EAP-36-e70166-s002.pdf]
