# Supplementary material for: Identifying the demographic pathways linking environmental covariates to population dynamics in an avian migrant
Source: Ecol Appl. 2026 Jan 5;36(1):e70166. doi: 10.1002/eap.70166 (PMC12770812; doi:10.1002/eap.70166)
Supplement: Supplementary file 13 — Appendix S13. [file EAP-36-e70166-s013.pdf]

Supporting information: Identifying the demographic pathways linking environmental covariates to population dynamics in an avian migrant

Appendix S13: Environmental covariates that had a significant effect on probability of fledging, continued from Discussion.

Section S1: Nest initiation date

For recruits, nests in years with earlier average nest initiation dates had a higher probability of fledging. Nest initiation dates are known to be important for breeding success, and there has been evidence of increased selection for earlier breeding in pied flycatchers (Both & Visser, 2001). This increased selection for earlier breeding could be attributed to deteriorating food availability during later dates in the breeding season (Perrins & Moss, 1975). For example, Siikamäki observed that chick weights in Pied Flycatchers were lower in later clutches compared to earlier ones, but supplemental feeding mitigated this effect (Siikamäki, 1996). Others have found that nests initiated early have higher reproductive success and larger clutch sizes in comparison to nests that are initiated later (Goodenough et al., 2009). Interestingly, this phenomenon was only found in nests from recruits, whose nest initiation dates averaged six days later than those of adults or immigrants over the course of the study (Appendix S1). It is possible that variation in nest initiation dates among adults and immigrants did not affect fledging success because, despite annual fluctuations, their nesting dates still aligned with optimal food availability periods. In contrast, recruits that were nesting on average six days later may be benefiting from earlier nest initiation, allowing them to take advantage of peak food availability that they may have previously missed.

Section S2: NDVI

Probability of fledging was lower for immigrant nests in years with higher NDVI (Figure 4A). Higher NDVI values translate into higher primary productivity, caused by warmer and moist conditions on the overwintering grounds. High NDVI values have been shown to influence

the availability of insects, ultimately being linked to increases in food availability (Buchan et al., 2021). Generally, high NDVI is considered a favorable winter condition for a songbird, and thus the negative relationship we found is surprising. In some populations of pied flycatchers, years of high NDVI on the overwinter grounds lead to early departure and concomitant early arrival onto the breeding grounds (e.g., Cotton, 2003). In our population in particular, this suggests that higher NDVI in winter can result in earlier departure from overwintering areas and consequently earlier arrival on the breeding grounds, but that this early arrival fails to translate into early nest initiation for immigrants (e.g. Balbontin et al. 2009, Both 2010, Finch et al. 2014, Robson & Barriocanal 2011). It is possible that we found the negative relationship with NDVI because immigrants failed to adjust the timing of nest initiation as well as adults and juveniles after a good NDVI year and thus had more competition for nest sites or miss peak food availability, which tends to be correlated with winter conditions.

Section S3: Mast Years

The probability of fledging was also lower for recruit nests in years following masting events. We used masting events as a proxy for dormice abundance, as dormice are breed in nest boxes and are known to reproduce in years with beech (*Fagus*) or oak (*Quercus*) mast seeding (Pilastro et al., 2003). Using mast years as a proxy, we show that dormice are detrimental to cavity-nesting birds such as pied flycatchers because they can depredate eggs and hatchlings during the late summer as well as occupy nest boxes, ultimately causing lower nest success. This result was also highlighted in another study system inclusive of pied flycatchers in the Czech Republic, where Adamík & Král, 2008 also noted that flycatcher nests were significantly more likely to be depredated than other cavity nesting species. They also noted that seasonally the latest breeding species had the highest brood losses of the species included

in their analysis (Adamík & Král 2008). Interestingly, the negative effect of mast events was only significant for recruit nests in our analysis. Dormice dormancy and sexual activity is directly linked to temperature (Wilz & Heldmaier, 2000), with higher temperatures serving as triggers for dormancy termination. Recruits in our study populations had on average later nest initiation dates than adults and immigrants and it is possible that therefore they were more likely to overlap with sexually active, nondormant dormice who are not active until relatively late in the breeding season due to thermal requirements (Juškaitis, 2006; Koppmann-Rumpf et al., 2003), implying that adult and immigrants avoid predation or competition with the dormice.

Section S4: Minimum temperature

Higher minimum temperatures during the hatchling period were associated with a higher probability of fledging for immigrant nests (Figure 4A). We show that in our population, it is not only average temperatures that are driving variation in probability of fledging (i.e., average summer temperatures), but also the minimum temperature extremes. This result is an extension of the known linear relationship between decreasing probability of nest success with decreasing temperatures in many passerine species. In pied flycatchers, hatchings are considered to be particularly temperature-sensitive (Shilov, 1973), and it is likely that in years with lower minimum temperatures hatchlings require more resources and are exposed to thermal stress due to cold snap periods, thus lowering their probability of fledging (Järvinen & Ylimaunu, 1986). We only found this effect in immigrant nests. Immigrant nests in our study site had on average four days earlier nest initiation dates than recruit nests. This means that it is possible that there were hatchlings in immigrants nests while there were still unhatched eggs in juvenile and recruit nests, benefitting from increased thermal protection from incubating parents and less affected by cold snaps.

References

Adamík, P., & Král, M. (2008). Nest losses of cavity nesting birds caused by dormice (Gliridae, Rodentia). *Acta theriologica*, *53*(2), 185-192.

Balbontín, J., Møller, A. P., Hermosell, I. G., Marzal, A., Reviriego, M., & De Lope, F. (2009). Individual responses in spring arrival date to ecological conditions during winter and migration in a migratory bird. *Journal of animal ecology*, *78*(5), 981-989.

Both, C. (2010). Flexibility of timing of avian migration to climate change masked by environmental constraints en route. *Current Biology*, *20*(3), 243-248.

Both, C., & Visser, M. E. (2001). Adjustment to climate change is constrained by arrival date in a long-distance migrant bird. *Nature*, *411*(6835), 296-298.

Buchan, C., J.J. Gilroy, I. Catry, J. Bustamante, A.D. Marca, P.W. Atkinson, J.M. González, & A.M.A. Franco (2021). Carryover effects of long-distance avian migration are weaker than effects of breeding environment in a partially migratory bird. *Scientific Reports* 11(1), 935.

Cotton, P.A. (2003) Avian migration phenology and global climate change. *Proceedings of the National Academy of Sciences of the United States of America*, **100**, 12219–12222.

Finch, T., Pearce-Higgins, J. W., Leech, D. I., & Evans, K. L. (2014). Carry-over effects from passage regions are more important than breeding climate in determining the breeding phenology and performance of three avian migrants of conservation concern. *Biodiversity and Conservation*, *23*(10), 2427-2444.

Goodenough, A. E., Elliot, S. L., & Hart, A. G. (2009). Are nest sites actively chosen? Testing a common assumption for three non-resource limited birds. *Acta Oecologica*, *35*(5), 598-602.

Järvinen, A., & Ylimaunu, J. (1986). Intraclutch egg-size variation in birds: physiological responses of individuals to fluctuations in environmental conditions. *The Auk*, 235-237.

Juškaitis, R. (2006). Interactions between dormice (Gliridae) and hole-nesting birds in nestboxes. *Folia Zool*, *55*(3), 225-236.

Koppmann-Rumpf, B., Heberer, C., & Schmidt, K. H. (2003). Long term study of the reaction of the edible dormouse Glis glis (Rodentia: Gliridae) to climatic changes and its interactions with hole-breeding passerines. *Acta Zoologica Academiae Scientiarum Hungaricae*, *49*(suppl 1), 69-76.

Perrins, C. M., & Moss, D. (1975). Reproductive rates in the great tit. *The Journal of Animal Ecology*, 695-706.

Pilastro, A., Tavecchia, G., & Marin, G. (2003). Long living and reproduction skipping in the fat dormouse. *Ecology*, *84*(7), 1784-1792.

Robson, D., & Barriocanal, C. (2011). Ecological conditions in wintering and passage areas as determinants of timing of spring migration in trans-Saharan migratory birds. *Journal of Animal Ecology*, *80*(2), 320-331.

Shilov, I. A. (1973). *Heat regulation in birds: An ecological-physiological outline*. Amerind.

Siikamäki, P. (1996). Nestling growth and mortality of Pied Flycatchers Ficedula hypoleuca in relation to weather and breeding effort. *Ibis*, *138*(3), 471-478.

Wilz, M., & Heldmaier, G. (2000). Comparison of hibernation, estivation and daily torpor in the edible dormouse, Glis glis. *Journal of Comparative Physiology B*, *170*(7), 511-521.
